# Ultrafast Switchable Polar and Magnetic Orders by Nonlinear Light–Matter Interaction

**DOI:** 10.1021/acsnano.5c06178

**Published:** 2026-08-13

**Authors:** Haoyu Wei, Daniel Kaplan, Haowei Xu, Ju Li

**Affiliations:** † Department of Applied Physics, 5755 Yale University, New Haven, Connecticut 06520, United States; ‡ Tsien Excellence in Engineering Program, Tsinghua University, Beijing 100084, China; § Center for Materials Theory, Department of Physics and Astronomy, Rutgers University, Piscataway, New Jersey 08854, United States; ∥ Department of Materials Science and Engineering, 2167 Massachusetts Institute of Technology, Cambridge, Massachusetts 02139, United States; ⊥ Department of Materials Science and Engineering, Massachusetts Institute of Technology, Cambridge, Massachusetts 02139, United States; # Department of Physics, City University of Hong Kong, Kowloon, Hong Kong SAR 999077, China; ¶ Department of Nuclear Science and Engineering, Massachusetts Institute of Technology, Cambridge, Massachusetts 02139, United States

**Keywords:** nonlinear light–matter interaction, ultrafast switching, sliding ferroelectricity, multiferroics, two-dimensional materials, quantum geometry

## Abstract

An outstanding challenge in materials science and physics is the harnessing of light for switching ferroic orders in, e.g., ferroelectrics. Here, we propose a mechanism through which electrons in few-layer 2D materials excited with above-bandgap light cause ionic structural transitions. Using perturbation theory within a many-body formalism, we show that the ionic coupling is mediated by a resonant change in electronic occupation functions, ultimately governed by the quantum geometric tensor (QGT) of the ground state. Furthermore, we show that such transitions are generally accompanied by multiferroic order switching. We demonstrate three examples of light-induced structural and polarization switching under this mechanism using first-principles calculations on bilayer CrI_3_, MoTe_2_, and trilayer 3R–MoSe_2_. We show that the three materials can switch between atomic stackings with a light intensity threshold of only ∼10^1^ GW/cm^2^, a value 2–3 orders of magnitude lower than that required by direct light-ion coupling thanks to the superior efficiency of resonant light-electron coupling. Since such switching is fast, highly controllable, contactless, and reversible, it is promising for use in optically controlled nonvolatile memory, nanophotonics, and polar electronic devices.

## Graphical abstract

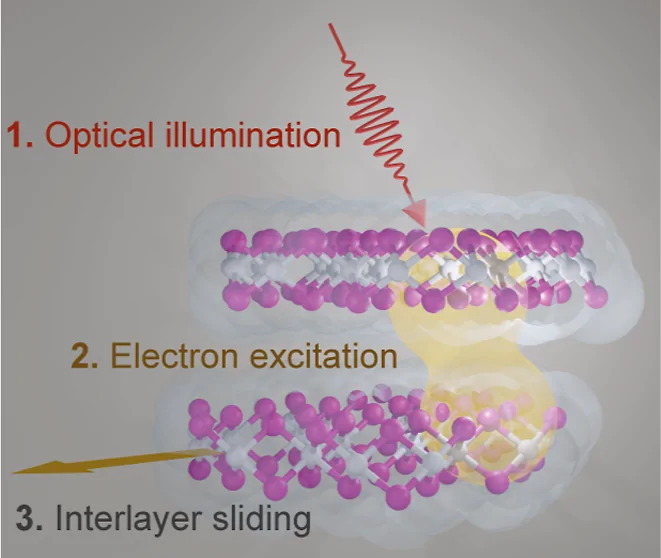

Switchable and reversible engineering of quantum materials with light is a long-sought goal of materials physics.^1–3^ A key ingredient for efficient switching is the existence of a manifold of states separated by energy gaps that can be overcome by the energy pumped with light. One common example of matter with competing orders with small gaps is the multiferroics.^4^ Over past decades, multiferroics have elicited extensive research interest for potential applications, with the ultimate goal of converting them into practical devices. The possibility of using multiferroics—and more specifically the diverse ferroic or magnetic orders found in them—as magnetically controllable ferroelectric memory, switches, and sensors has been proposed^5^ and is the subject of immense experimental effort. Recent work has explored the possibility that competing ferroic orders can be used as a switch. For instance, ferroelastic transitions have been proposed theoretically^6^ and supported experimentally^7^ in the 1T-to-1T′ transition of monolayer transition-metal dichalcogenides (TMD). Ferromagnetism has also been seen in a range of 2D materials including CrI_3_,^8^ Cr_2_Ge_2_Te_6_,^9^ and CrXBr.^10–13^ Moreover, an inversely stacked CrI_3_ has been predicted and shown to also be ferroelectric, realizing a multiferroic state.^14,15^ Beyond intrinsic properties of 2D stacks, experimentally observed sliding ferroelectricity^16,17^ offers a way of realizing tunable out-of-plane ferroelectricity in bilayers that is switched by interlayer shifts of the 2D constituents composing the structure.^18^

The use of light to tune between and induce multiferroic behavior locally, reversibly, and in a switchable manner is particularly attractive. To achieve this, several reports considered coupling via phonons,^19,20^ thermal effects,^21^ or direct light-electron interactions, with the latter primarily focusing on nonsoft, sliding transitions, such as optical tuning of AB stacked h-BN^22^ and SnO.^23^ Light-induced magnetization reversal was separately considered in CrI_3_.^24,25^ However, these works neglected contributions arising from quantum coherence and focused only on the classical dielectric response. While structural transitions driven by electronic pumping have been empirically observed,^26,27^ the underlying physical mechanism remains unclear, and a unified theoretical framework connecting electronic and structural transitions is still lacking.

In this work, we systematically analyze the effects of above-bandgap optical light on ions using perturbation theory from a many-body formalism, a theoretical framework we call Nonlinear Electron-mediated Light-Ion Coupling (NELIC). Using NELIC, we demonstrate that resonant visible light, by inducing interband electronic transitions, can drive structural transitions. To demonstrate this effect, we focus on sliding transitions in two-dimensional (2D) materials primarily due to the low energy barrier. We concretely show light induces a sliding structural transition and hence switches the polarization in bilayer CrI_3_, MoTe_2_, and trilayer 3R–MoSe_2_—these vdW heterostructures^15,28–35^ have been shown to exhibit robust ferroelectricity. We also discuss detailed criteria for finding such switching in more general van der Waals (vdW) materials.

Schematically, our physical mechanism is described in Figure 1b. The free energy in equilibrium is symmetric between two states (two atomic stackings, as shown in Figure 1c). Upon illumination by above-bandgap light (curves of different colors in Figure 1b), the double-well free energy deforms and softens into a single well, causing instability in the atomic structure that leads to sliding. The control over which stacking softens depends on the energetics and matrix elements of the electronic states (and their associated quantum geometry), as shown in Figure 1a.

**Figure 1:**
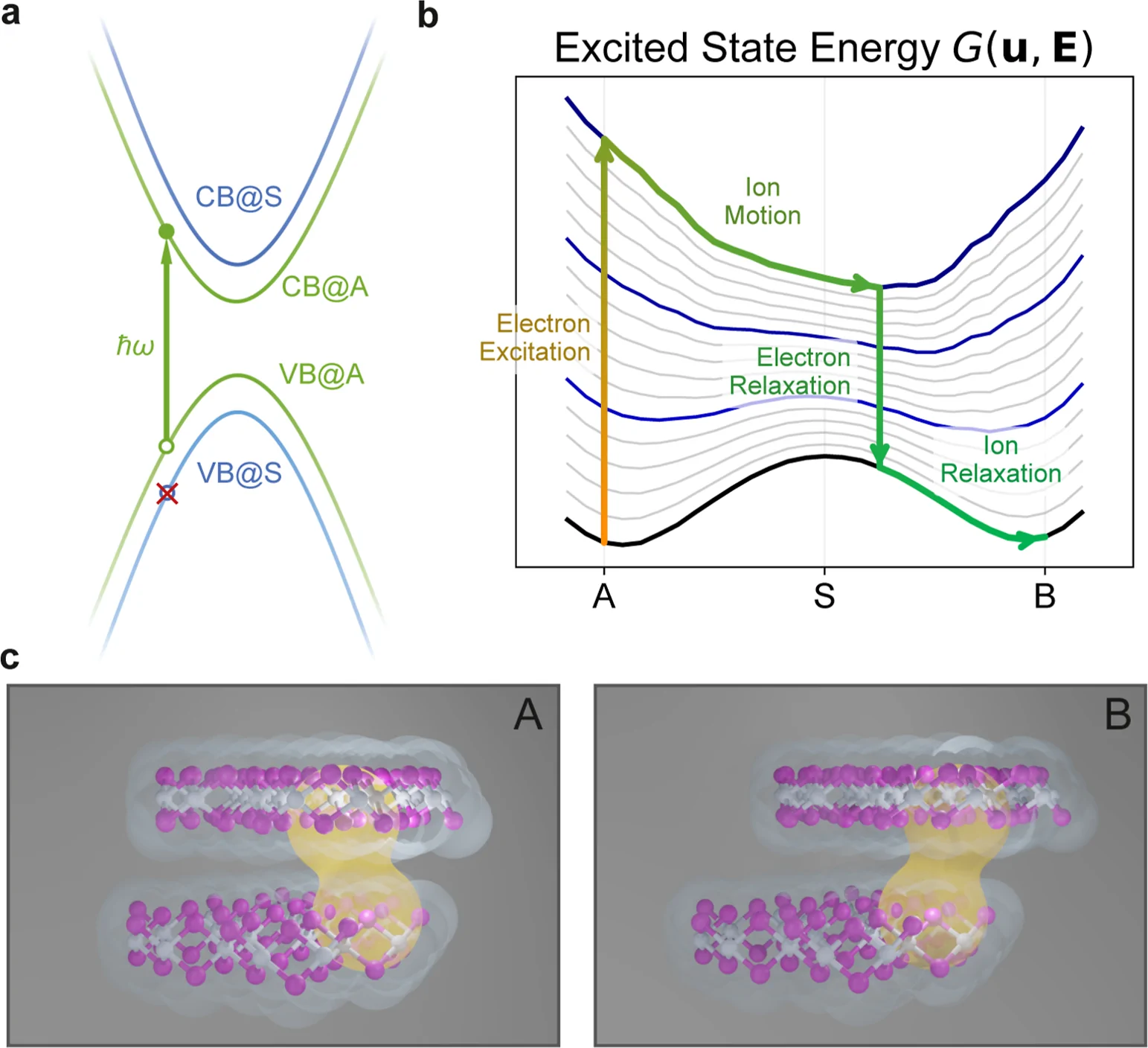
Principles of above-bandgap light-driven ferroic order switching. (a) Light generates excited states dependent on ionic order. CB, conduction band; VB, valence band. (b) The system’s Born–Oppenheimer (BO) surface changes as the system is excited from the ground state (black curve in the bottom) to excited states (gray and blue curves), following the path indicated by arrows in orange. (c) Two stackings of bilayer CrI_3_ that can switch to each other by sliding under light pumping (excited electron cloud schematically shown in yellow). In (a–c), A, B, labels for two locally stable ionic orders; S, label for the ionic order at saddle point in the switching path.

Using NELIC with parameters from first-principles calculations, we find that specifically chosen light frequency and polarization can independently and reversibly trigger a multiferroic transition in these materials. For bilayer *M*_z_-*T*_d_-MoTe_2_ and trilayer 3R–MoSe_2_, we demonstrate ferroelastic (sliding) switching, concomitant with ferroelectric switching, via the NELIC light-electron–ion pathway. In CrI_3_, besides ferroelastic and ferroelectric switchings, we additionally trigger its ferromagnetic order switching separately from ferroelastic switching, through the nonlinear Edelstein effect.^24^ Unifying the two mechanisms, we eventually demonstrate that the ferroelastic and ferromagnetic effects are governed by contrasting elements of quantum geometry.

Our findings establish a new method of controlling crystal structure with optics, which is readily observable in experiments, leading to ultrafast, reversible, and widely applicable switching of electronic and ionic order in solids.

## Results and Discussion

### Theory of Nonlinear Electron-mediated Light-ion Coupling

We begin by introducing the theoretical framework of Nonlinear Electron-mediated Light-Ion Coupling (NELIC). Under an incident electric field **E**(*t*) = **E**_0_ cos ω*t*, the free energy *G* of an electron–ion system is given by

1 G(u,E)=Φ(u,E)−E·p(u)

where **p** is the dipole moment of the electron–ion system, and **u** denotes the positions of all ions **u** = {**u**_*i*_}. Here, Φ­(**u**, **E**) is the Born–Oppenheimer (BO) energy surface for a given ionic configuration and electric field **E**. The free energy *G*(**u**, **E**) will thus be the effective potential felt by the ionic degrees of freedom, which in turn drives ionic transitions. This treatment is consistent with the Born–Oppenheimer approximation,^36,37^ as further justified in the Supporting Information, Section S14. In the rest of this section, we will derive an expression for *G*(**u**, **E**) as a function of input field and ground-state material properties, by careful evaluation of each constituent term from a many-body perspective, to arrive at eq 7.

While the free energy *G*(**u**, **E**) generally contains many harmonics of ω due to the nature of nonlinear response, only its static component will be relevant for significant ionic motion, as ionic motion frequencies are typically 10^–2^–10^–3^× lower than optical frequencies ω at which electrons are resonant. (In Supporting Information, we quantitatively compare and find other competing terms to be subdominant.) The effect of the static component accumulates in time, resulting in ionic motion.

Working in the Kohn–Hohenberg formalism of density functional theory (DFT),^38–40^ we express the first term in eq 1 using its density-related constituents

2 $$\begin{array}{l} \Phi \left(u,E\right)=T_{e}\left[\rho \left(r,u,E\right)\right]+V_{e-e}\left[\rho \left(r,u,E\right)\right]+\\ \sum_{i}\int \frac{-Z_{i}e^{2}\rho \left(r,u,E\right)}{\left|r-u_{i}\right|}dr+\sum_{i>j}\frac{Z_{i}Z_{j}e^{2}}{\left|u_{i}-u_{j}\right|} \end{array}$$

Here, *T*_*e*_(ρ) is the kinetic energy density functional, and *V*_*e*–*e*_ is the electron–electron interaction functional. ρ­(**r**, **u**, **E**) is the many-body electronic density as a function of local coordinate **r** and electric field **E**. The summation indices *i*, *j* refer to ionic positions. The third term, $V_{e-i}=\sum_{i}\int \frac{-Z_{i}e^{2}\rho \left(r,u,E\right)}{\left|r-u_{i}\right|}dr$ is the ion-electron coupling that, as we see later, feeds back the effect of light onto the ionic motion. Applying the Kohn–Sham approximation,^38^ Φ­(**u**, **E**) can be evaluated using single-electron Hamiltonian in the Bloch basis *H*|*u*_*n* **k**_⟩ = ℏω_*n* **k**_|*u*_*n* **k**_⟩ of the solid

3 $$\Phi \left(u,E\right)=T_{e}+V_{e-e}+V_{e-i}+V_{i-i}=Tr\left(\rho H\right)=\sum_{n}\int_{k}\rho_{nk}\hbar \omega_{nk}$$

where ρ_*n* **k**_ is the density matrix diagonal element of the *n*-th Bloch band at momentum **k**. We use the shorthand $\int_{k}\equiv \frac{1}{{\left(2\pi \right)}^{d}}\int d^{d}k$ where *d* is the spatial dimension.

As we detail in the Supporting Information, by expanding Φ­(**u**, **E**) to leading order in **E** in a many-body formalism, the unperturbed BO energy surface in equilibrium Φ­(**u**, 0) is affected at finite **E** purely by a change in zero-frequency electronic occupation. We find that

4 $$\Phi \left(u,E\right)=\Phi \left(u,0\right)+\sum_{n}\int_{k}\left\langle \delta \rho_{nk}\left(E\right)\right\rangle \hbar \omega_{nk}$$

where ⟨·⟩ signifies time-averaging, and

5 $$\left\langle \delta \rho_{nk}\left(E\right)\right\rangle =\frac{ie^{2}}{\hbar^{2}\omega^{2}\gamma_{nn}}\sum_{lab}\frac{f_{nl}v_{\ln}^{a}v_{nl}^{b}}{\omega_{\ln}-\omega -i\gamma_{\ln}}E_{a}\left(\omega \right)E_{b}\left(-\omega \right)+c.c$$

Equation 5 represents the first nonvanishing term in the perturbation theory of static change in Bloch state occupation number due to light illumination, which can be derived by perturbatively solving the electron master equation under light as we detail in Supporting Information. γ_ln_ is the electronic decay rate from level *l* to *n*, ω is the frequency of light. Here, $v_{\ln}^{a}\equiv \left\langle u_{lk}\left|{\mathrm{v̂}}^{a}\right|u_{nk}\right\rangle$ is the velocity operator in the *a*(=*x*, *y*, *z*) direction evaluated under Bloch basis, and we use the shorthands *f*_ln_ ≡ *f*_*l* **k**_ – *f*_*n* **k**_ for the equilibrium occupation number difference between bands *l*, *n*, and ω_*nl*_ ≡ ω_*n* **k**_ – ω_*l* **k**_ is the resonant frequency between bands.

For the second term in eq 1, that is, the linear charging of the dielectric, insulating medium, we can write it in terms of the dielectric response function χ^*ab*^(ω) as

6 $$-E\cdot p\left(u,E\right)=-\frac{{V_{u.c.}\epsilon }_{0}}{2}\sum_{ab}R\left(\chi^{ab}\left(\omega \right)E_{a}E_{b}^{*}\right)$$

where *V*_u.c._ is the unit cell volume. This term however is subleading in determining the free energy change Δ*G*(**u**, **E**) for above-bandgap light, which we argue as follows: assuming a characteristic density of states in the valence band *D*, velocity *v*, and relaxation time γ_ln_^–1^ ∼ τ, the occupation energy change expressed through eqs 4 and 5 takes the order of magnitude given by $\omega \tau \frac{e^{2}v^{2}V_{u.c.}DE^{2}}{\hbar \omega^{2}}$ , while the charging energy eq 6 is of order $\frac{e^{2}v^{2}V_{u.c.}DE^{2}}{\hbar \omega^{2}}$ . As the ratio between them is ∼ωτ ≫ 1, we safely neglect corrections from dielectric charging (eq 6). This distinction of relative scale is further demonstrated numerically in Supporting Information. Finally, combining eqs 4 and 5, the total change in the free energy (per unit cell) can be expressed as

7 $$\begin{array}{l} G\left(u,E\right)-G_{0}\left(u\right)\\ =\frac{ie^{2}V_{u.c.}}{\hbar \omega^{2}}\sum_{nl\omega ab}\int_{k}\left(\frac{\omega_{n}}{\gamma_{nn}}-\frac{\omega_{l}}{\gamma_{ll}}\right)\frac{f_{nl}v_{nl}^{a}v_{\ln}^{b}}{\omega_{nl}-\omega -i\gamma_{nl}}E_{a}\left(\omega \right)E_{b}\left(-\omega \right) \end{array}$$

for above-bandgap light. Equation 7 is the main result of our work, and enables the calculation of the correction to the free energy for a given ionic configuration **u** under an applied electric field **E** using the static, equilibrium Bloch states *n*, *l*.

We note several features of eq 7. First, this response function defined by a rank-2 symmetric tensor ${\mathrm{\chi ̃}}_{ex}^{ab}=\frac{\partial^{2}G\left(u,E\right)}{\partial E_{a}\partial E_{b}^{*}}$ is symmetric (even) under time-reversal, spatial inversion, and complex conjugation, consistent with physical constraints and Kramers–Kronig relations (see Supporting Information). It is generically nonvanishing for any point group symmetry, making this NELIC effect broadly relevant. Second, the numerator in eq 7 is proportional to matrix elements of Bloch velocity operators, which encode the quantum geometric properties of the solid.^41,42^ Third, if we adopt the simple relaxation time approximation γ_ln_ = τ^–1^ independent of band energy or momentum, this nonlinear response eq 7 can be connected to the linear optical conductivity through a fluctuation–dissipation relation (see Supporting Information).

Generally, NELIC response eq 7 produces a varying excitation energy as a function of ionic structure, changing the BO surface landscape. Since this function also depends on the frequency and polarization of light, we show in the following two sections that controlling these light parameters can result in bidirectional switching of the multiferroic order in 2D materials.

### Multiferroic Transition in Bilayer CrI_3_ and *T*_d_-MoTe_2_

As a central prediction of NELIC, eq 7 allows for the calculation of *G*(**u**, **E**) in the presence of arbitrary symmetry. We now show that in the presence of local minima in the free energy, specifically in the low-barrier interlayer sliding of vdW 2D materials, NELIC enables light-induced switching between these local minima states as well as the concomitant ferroic orders if present.

To construct the bilayer, we stack monolayers such that inversion symmetry is not restored when both are within the same unit cell, to ensure ferroelectricity and to avoid degenerate ground states, for reasons that will become clear later and also detailed in Supporting Information Section S8. This can be done in the vdW/TMD context by applying the *M*_*z*_ operation on one layer, consistent with the prescription in ref 14 that enables ferroelectricity. Now, from this base structure where top and bottom layers are laterally aligned, a stacking configuration **u** is then formed by shifting one layer with respect to another by (*x*, *y*) → (*x* + *au*_*x*_, *y* + *au*_*y*_) where *a* is the lattice constant. Such structures, as in the case of hBN and CrI_3_ ^43^, have been realized experimentally.

Constructing bilayers from CrI_3_ and MoTe_2_ in the manner described above (see Supporting Information Sections S5 and S6 for details), we now show that NELIC allows for switching between different ferroic orders therein. Using the PBE-GGA functional^44^ within DFT as implemented in the Vienna ab initio simulation package (VASP),^45,46^ we compute the free energy *G*(**u**, **E** = **0**) for every stacking **u** (see Methods for computational details). We plot this free energy landscape for CrI_3_ in Figure 2a, where we find two nonequivalent local minima $A\left(0,\frac{1}{3}\right)$ and $B\left(\frac{1}{3},\frac{1}{3}\right)$ separated by energy barrier of 25 meV at saddle point *S*. The two types of local minima *A*, *B* separated by *E*_*B*_ – *E*_*A*_ = 1.6 meV are not related by any symmetry, allowing optical switching between them. Between them, we extract a minimal energy path (MEP), which we highlight by an orange line on Figure 2a.

**Figure 2:**
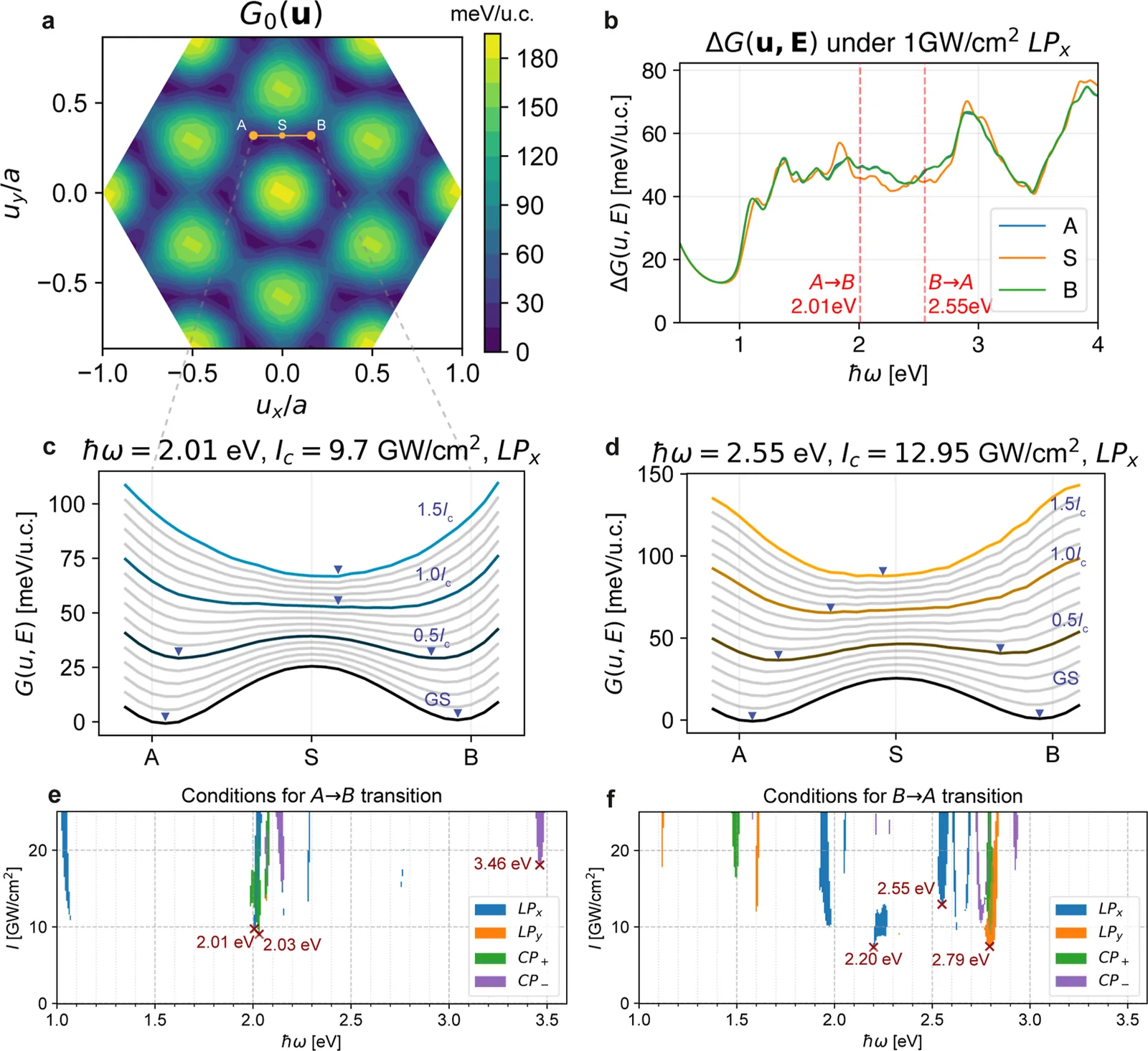
Light-induced sliding in bilayer *M*_*z*_-CrI_3_. (a) Equilibrium energy of bilayer *M*_*z*_-CrI_3_ of different stackings parametrized by **u** = (*u*_*x*_, *u*_*y*_), the in-plane relative translation vector of one layer. Indicated as a yellow line is an MEP between two locally stable stackings A and B, through a saddle point *S*. (b) The nonlinear excitation free energy under 1 GW/cm^2^ *LP*_*x*_ in the three key stackings *A*, *S*, and *B*. Differences in the excitation energies between stackings are utilized to induce sliding. (c) (d) As pumping light intensity increases from 0 (ground state, GS) to 0.5, 1, 1.5 times the respective critical value, a structural transition in both directions can be induced at 2.01 eV (c, from *A* to *B*) and 2.55 eV (d, from *B* to *A*). (e) (f) The suitable light conditions for bidirectional switchings. The frequency and intensities that can trigger *A* → *B* and *B* → *A* switching are filled in colors indicating the polarization of light. Hybrid colors that indicate more than one polarization can induce switching. *LP*_*x*_(*LP*_*y*_), linear polarized light along *x* (*y*) direction; *CP*_+_(*CP*_–_), left- (right-) circular polarization.

From the DFT calculations, we then perform a Wannier interpolation of electronic states for every stacking **u**,^47,48^ and evaluate the frequency-dependent free energy Δ*G*(**u**, **E**(ω)) along the MEP, showing it at the special points *A*, *S*, and *B* in Figure 2b. This function’s slight but nonvanishing dependence on **u**, originating from vdW interaction-induced electron density reconfiguration, offers us the opportunity to bias the free energy toward certain stackings and thus trigger structural transitions.

Specifically, as shown in Figure 2c, we find that under ℏω = 2.01 eV, as light intensity increases, the free energy curve *G* softens and biases toward *B*, before fully transforming to a single-well potential, with a single local minimum marked by the blue arrows within the *S*–*B* region, above a critical intensity *I*_*c*_ = 9.7 GW/cm^2^. This allows us to achieve a full *A* → *B* structural transition through a path illustrated in Figure 1b: a structure initially at *A* can be excited to a higher BO surface under light, which biases it across the ground state saddle point *S*. Then, when electrons fully relax after light is removed and BO surface is recovered, the ionic structure will fully relax to *B*. Conversely, we show in Figure 2d that under light frequency *ℏ*ω = 2.55 eV, the structure can be shifted in the inverse direction *B* → *A*, following the same procedures of electronic excitation, ionic motion, electronic relaxation, and finally ionic relaxation. Beyond the two examples, we systematically scan through the light-parameter space spanned by frequency, intensity, and polarization, and detect the regimes that enable switching, as shown in Figure 2e,f. The resulting condition map exhibits extended regions, indicating that the proposed switching mechanism is robust against variations in frequency and intensity.

Notably, this controllable light-induced sliding­(ferroelastic switching), is often accompanied by a ferroelectric switching, because different slidings can exhibit different polarization strengths and directions due to vdW-induced charge redistribution in the bilayer.^14,16,49,50^ In Figure 3a, we calculate the polarization *z*-component *P*_*z*_ from DFT charge densities at different stackings, revealing an alternating pattern in the sliding position space, which is governed by several symmetries:^14,51^ *P*(*C*_2*z*_ **u**) = – *P*(**u**), *P*(*m*_110_ **u**) = *P*(**u**), and $P\left(m_{11̅0}u\right)=-P\left(u\right)$ . Importantly, we see that stackings *A* and *B* have opposite polarizations, implying that NELIC-induced sliding between them is necessarily accompanied by an out-of-plane ferroelectric dipole flip.

**Figure 3:**
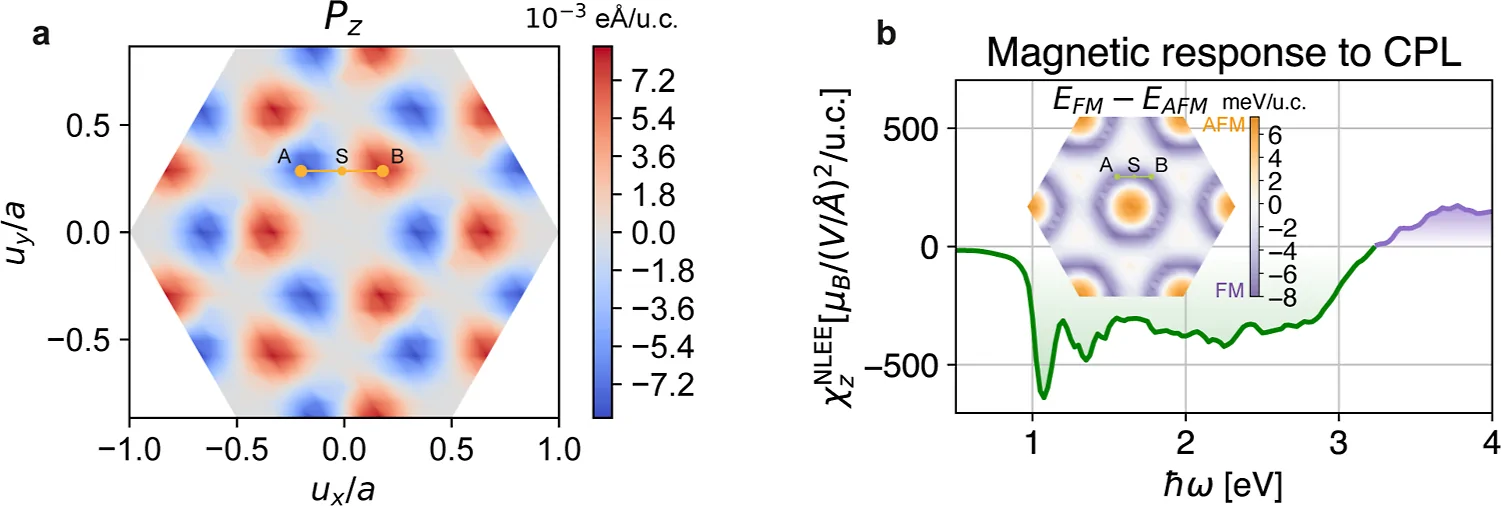
Multiferroic transition in *M*_*z*_-CrI_3_ induced by nonlinear interaction. (a) Out-of-plane stacking-dependent ferroelectric polarization dipole in the ground state, also showing the same transition path A–S–B marked in Figure 2a. The alternating polarization pattern allows ferroelectric order flipping along with the structural transition. (b) At stacking *A*, the magnetization generated by Nonlinear Edelstein Effect (NLEE) as a function of incident circularly polarized photon energy. Inset shows the DFT energy difference *E*_FM_ – *E*_AFM_, indicating that ferromagnetic order is preferable throughout the transition path *A*–*S*–*B*.

A qualitatively similar picture is found for *M*_*z*_-*T*_*d*_-MoTe_2_, whose ground state stacking energy is plotted in Figure 4a. Here, the two local energy minima $A\left(\frac{1}{2},0\right)$ and $B\left(\frac{1}{4},\frac{1}{2}\right)$ have a much larger energy difference compared to CrI_3_. Evaluating *G* for stackings along an approximate MEP under two representative frequencies in Figure 4c,d), our results show controllable bidirectional switching *A* ↔ *B* under 3.48 and 1.94 eV in a similar manner. We also identified a much richer polarization landscape in bilayer MoTe_2_ as shown in Figure 4b, owing to its lower point group symmetry. As a result, *A* ↔ *B* sliding is accompanied by polarization *P*_*z*_ switching between *P*_*z*_(*A*) = 0 and *P*_*z*_(*B*) = 10.5 × 10^–3^ eÅ/u.c, thereby representing a paraelectric-to-ferroelectric transition.

**Figure 4:**
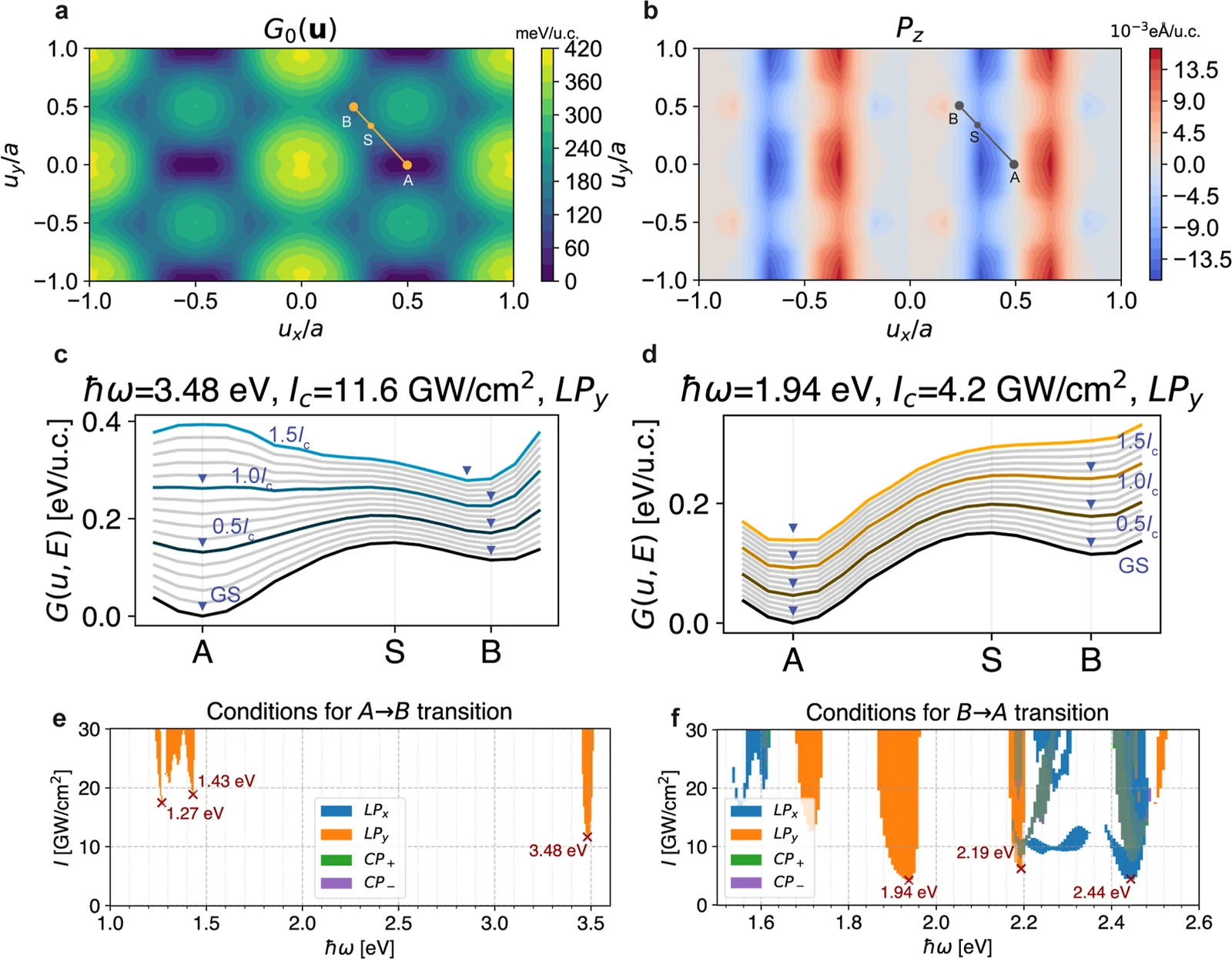
Light-induced sliding in bilayer *M*_*z*_-*T*_*d*_-MoTe_2_. (a) Ground state energy and (b) out-of-plane polarization of *M*_*z*_-*T*_*d*_-MoTe_2_, with transition path A–S–B indicated. (c,d) Excited state energy under two different frequencies of pumping light of increasing intensities, used to induce (c) A–B transition and (d) B–A transition. Curves show free energy as light intensities are increased from 0 (ground state, GS) to 0.5, 1.0, 1.5 times the respective critical intensity for transition. (e,f) The suitable light conditions for bidirectional switchings. The frequency and intensities that can trigger A → B and B → A switching are filled in colors indicating the polarization of light. Hybrid colors indicate more than one polarization can induce switching. *LP*_*x*_(*LP*_*y*_), linear polarized light along *x* (*y*) direction; *CP*_+_(*CP*_–_), left- (right-) circular polarization.

Independent from the ferroelastic and ferroelectric orders discussed above, in layers with a concomitant ferromagnetic (FM) order, it is also possible to flip its direction via nonlinear Edelstein effect (NLEE),^24,25^ which relies on direct coupling of light’s angular momentum to electrons and is insensitive to stacking. Here, we compute the NLEE coefficient on *M*_*z*_-CrI_3_, including both spin and orbital contributions, at stacking *A* using the same method as in ref 24 and plot its frequency dependence in Figure 3b. Abiding by the same criterion as ref 24, we note that at frequencies between 1.0 and 2.7 eV, light intensity above μ_*B*_/χ_*z*_^NLEE^ ∼ 33 GW/cm^2^ can induce magnetization δ*M*_*z*_ comparable to the intrinsic magnetization ∼μ_B_, thereby inducing magnetic switching. The direction of induced magnetization will depend strongly on the chirality of light, enabling polarization control of magnetization flipping.

Importantly, because ferroelastic/ferroelectric switching and NLEE-driven magnetic switching originate from distinct physical mechanisms with different polarization and frequency selectivities, these order parameters can be controlled largely independently: sliding-induced ferroelastic and ferroelectric switching can be driven by either circularly polarized light (CPL) or linearly polarized light (LPL), while ferromagnetic switching is primarily controlled by CPL, with the frequency providing an additional tuning knob for selectivity. Together, these features establish the proposed scheme as a genuinely multiferroic optical-control platform.

### Multiferroic Transition in Trilayer 3R–MoSe_2_

To demonstrate the generality of NELIC-induced multiferroic switching, here we also demonstrate such transitions in a trilayer system MoSe_2_ within a substrate electric field. A standalone trilayer MoSe_2_ has two locally stable stackings “ABC” (hereby labeled as *A*) and “CBA” (hereby labeled as *B*) which can be seen as the laterally aligned trilayer being oppositely shifted. To consider sliding between these two states, we include the space of all oppositely shifted trilayers while leaving out other possible trilayers for simplicity, i.e., whenever the bottom layer is shifted by a vector **u**_*b*_, we shift the top layer by **u**_*t*_ = −**u**_*b*_, and keep the middle layer fixed. We appoint “ABC” (*A*) as the base structure (**u**_*b*_ = **u**_*t*_ = 0), a definition different from the convention for the bilayers above but does not affect the physics (see details in Figure S3). What is special about this structure is that, because these two stackings *A* and *B* are related by an out-of-plane mirror symmetry *M*_*z*_, they respond identically under normally incident light according to eq 7, which cannot lead to switching. To break this symmetry, we assume a substrate generating a uniform + *z* static electric field of 0.005 V/Å^52^ across the MoSe_2_ structure. Doing this allows us to observe a similar NELIC-induced sliding behavior coupled with ferroelectric order switching across the MEP, shown in Figure 5.

**Figure 5:**
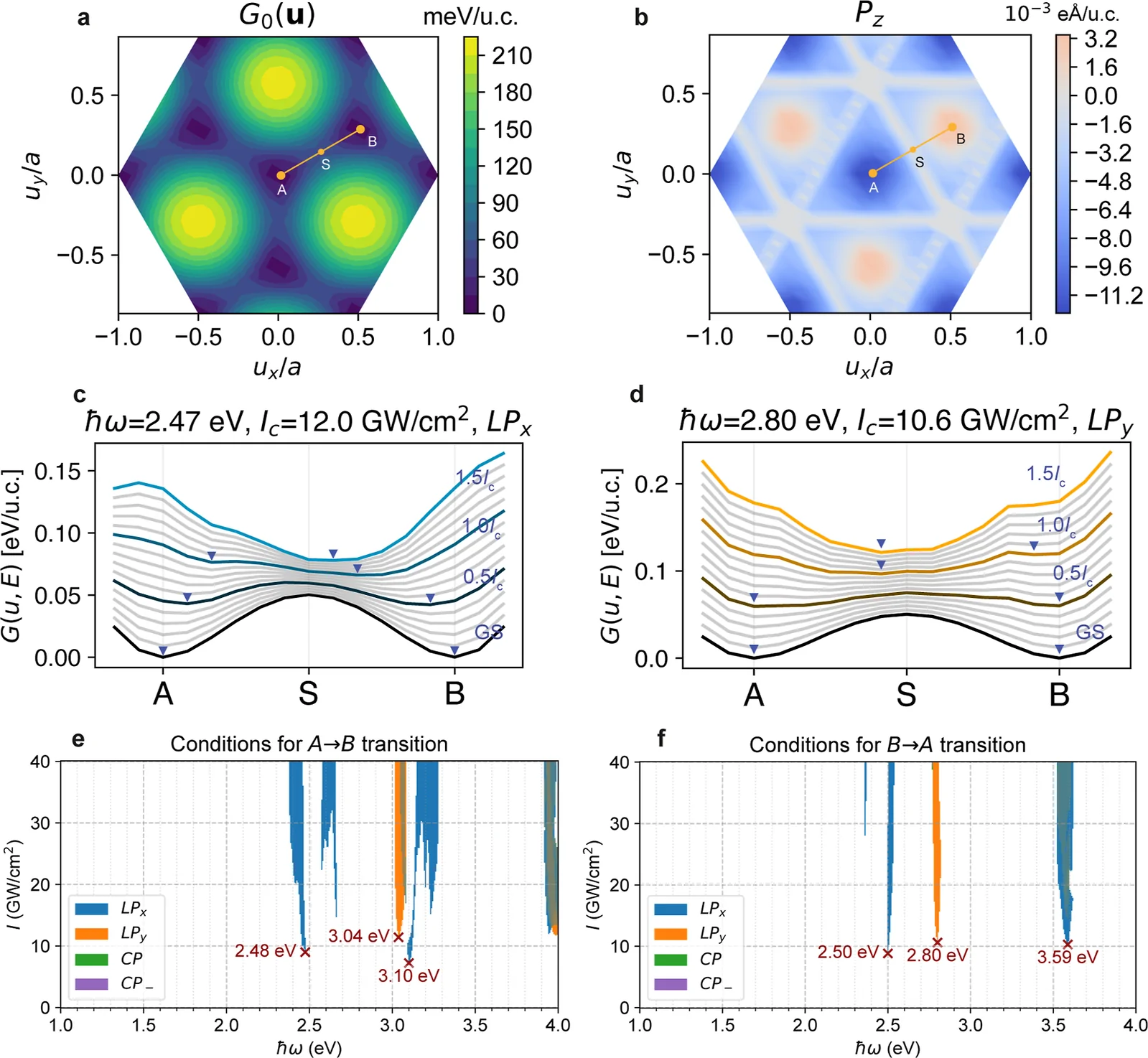
Light-induced sliding in 3R–MoSe_2_ on a substrate. (a) Ground state energy and (b) out-of-plane polarization of 3R–MoSe_2_, with transition path A–S–B indicated. (c,d) Excited state energy under two different frequencies of pumping light, used to induce (c) A–B transition and (d) B–A transition. Curves show free energy as light intensities are increased from 0 (ground state, GS) to 0.5, 1.0, 1.5 times the respective critical intensity for transition. (e,f) The suitable light conditions for bidirectional switchings. The frequency and intensities that can trigger A → B and B → A switching are filled in colors indicating the polarization of light. Hybrid colors indicate more than one polarization can induce switching. *LP*_*x*_(*LP*_*y*_), linear polarized light along *x* (*y*) direction; *CP*_+_(*CP*_–_), left- (right-) circular polarization.

To generalize this symmetry consideration to other 2D materials, we note that while NELIC-induced sliding is broadly applicable, symmetry constraints can preclude switching by enforcing equivalent responses at different stackings. We summarize guidelines for identifying viable switching candidates (see Supporting Information Section S8 for details):

1. Analyze Stacking Symmetries: Switching requires nonequivalent NELIC responses along the MEP. Symmetry analysis can identify degenerate stackings with equivalent responses but alone cannot predict the existence of additional nonequivalent metastable states essential to switching.
2. Calculate stacking energies via DFT: Compute the stacking energy landscape (e.g., Figure 2a) to identify nonequivalent metastable states as potential switching end points.
3. Combining Symmetry and DFT Results: Apply the symmetry analysis from step 1 to the metastable stackings identified in step 2. If two metastable stackings are not related by symmetry, switching between them is generally achievable with an appropriate light frequency and polarization.

### Quantum Geometry of Light-Induced Sliding

As shown, eq 7 is intimately connected to the matrix elements of Bloch states, defining quantum geometry.^53–56^ We use the property that *v*_*nl*_^*a*^ = *iω*_*nl*_ *r*_*nl*_^*a*^, where *r*_*nl*_^*a*^ = ⟨*u*_*n* **k**_|∂_*k*_*a*__|*u*_*l* **k**_⟩ is the relation between the velocity operator and the generally (non-Abelian) position operator. Working in the relaxation time approximation and assuming ωτ ≫ 1, ω_*nl*_ = ω. We recast eq 7 as

8 $${\mathrm{\chi ̃}}_{ex}^{ab}=\frac{\partial^{2}G\left(u,E\right)}{\partial E_{a}\left(\omega \right)\partial E_{b}\left(-\omega \right)}=\frac{V_{u.c.}\pi e^{2}\omega \tau }{\hbar }\sum_{nl}\int_{k}f_{nl}r_{nl}^{a}r_{\ln}^{b}\delta \left(\omega -\omega_{\ln}\right)$$

We recognize the quantity *r*_*nl*_^*a*^ *r*_*ln*_^*b*^ as the quantum geometric tensor *Q*_*nl*_^*ab*^ = *g*_*nl*_^*ab*^ + *i*Ω_*nl*_^*ab*^/2.^57^ Here, *g*_*nl*_^*ab*^ is the (non-Abelian) quantum metric for bands *n*, *l* while Ω^*ab*^ is their Berry curvature. Unsurprisingly, this quantity governs the entirety of the coupling to the electric field, as argued in the past,^42,58^ since *g* ^*ab*^ is symmetric in *a*, *b*, it is primarily responsible for linear-polarized light coupling while Ω^*ab*^ being antisymmetric couples only to circularly polarized light. However, it is Ω^*ab*^ that controls an induced magnetic moment in the NLEE,^24^ unifying the ionic transition with the electronic one. We note an important consequence of this observation: ref 59 introduced the time-dependent quantum geometric tensor (tQGT) as

9 $$Q^{ab}\left(t-t^{\prime }\right)=\sum_{nl}f_{n}\left(1-f_{l}\right)e^{i\omega_{nl}\left(t-t^{\prime }\right)}r_{nl}^{a}r_{\ln}^{b}$$

Following ref 59, the physically relevant quantity is the antisymmetric part of this tensor, i.e., *Q*_asym._^*ab*^(*t*) = (*Q* ^*ab*^(*t*)–*Q* ^*ba*^(−*t*))/2.

Examining the ω harmonic of *Q*_asym._^*ab*^(*t*) we find, for insulators, (setting *t* −*t*′ → *t*)­

10 $$\begin{array}{ll} Q_{asym.}^{ab}\left(\omega \right) & =\sum_{nl}f_{n}\left(1-f_{l}\right)[\delta \left(\omega -\omega_{\ln}\right)r_{nl}^{a}r_{\ln}^{b}-\\ & \delta \left(\omega -\omega_{nl}\right)r_{nl}^{b}r_{\ln}^{a}]=\\ & \sum_{nl}f_{nl}\delta \left(\omega -\omega_{\ln}\right)r_{nl}^{a}r_{\ln}^{b}, \end{array}$$

identical with the matrix elements appearing in eq 8. Likewise, we note that for NLEE,^24^ the response tensor is equal to $\xi_{a,b}^{z}=\pi \tau \frac{e^{2}}{2\hbar^{2}}\int_{k}f_{nl}\left(r_{nl}^{a}r_{lm}^{b}-r_{nl}^{b}r_{\ln}^{a}\right)\left(L_{n}-L_{l}\right)\delta \left(\omega -\omega_{nl}\right)$ . If, additionally, the electronic bands have well-defined and **k**-independent angular momentum, **L**_*n*_ (as near the band edges in CrI_3_), the above expression can be simplified to $\xi_{a,b}^{z}=\pi \tau \frac{e^{2}\Delta L}{2\hbar^{2}}\int_{k}Im\left(Q_{asym.}^{ab}\left(\omega \right)\right)$ . All calculations were carried out using the full expression for ξ_*z*_, which includes the momentum dependence of Δ*L*. Thus, we establish a link between light-induced Fermi excitations, eq 5, and the frequency harmonics of the tQGT, making light-induced structural and magnetic transitions a sensitive, resonant probe of quantum geometry. As shown in Figure S8 (see Supporting Information), the transitions between states which enable the sliding in CrI_3_ occur between Cr orbitals of strongly hybridized bands. In a similar vein, a recent proposal showed strongly hybridized bands with inversion symmetry breaking can produce out-of-plane polarization in superconductors.^60^ The change in electronic occupation can be related to gravitational anomalies in solids, closely connected with quantum geometry.^61,62^

### Experimental Considerations

Here, we provide further evidence suggesting that this proposed effect is readily observable in an experiment without compromising the sample. In Supporting Information Sections S13–S15, we show that our NELIC framework can be extended to the cases of short pulses by replacement of the factor *I*τ with fluence *F* = *I* × *t*_pulse_ in eq 7. Accordingly, the resulting pulse requirements are well within experimentally achievable regimes. In particular, comparable fluences and associated temperature rises have been reported in prior experiments without inducing sample degradation. Here, we summarize our proposed light conditions, along with a comparison to representative experimental benchmarks, into Table 1.

**Table 1:** Expected Threshold Intensity (Under Continuous Wave, CW), Fluence Under Pulses, and Estimated Lattice Temperature Rise for Structural Transitions Considered, With References to Representative Ultrafast Optical Experiments on the Same Materials

material	*M*_*z*_-CrI_3_		*M*_*z*_-*T*_*d*_-MoTe_2_		3R–MoSe_2_	
switching	A → B	B → A	A → B	B → A	A → B	B → A
photon energy (eV)	2.01	2.55	3.48	1.94	2.47	2.80
wavelength (nm)	617	486	356	639	502	443
intensity (CW) (GW/cm^2^)	9.7	12.95	11.6	4.2	12.0	10.6
fluence (pulsed) (μJ/cm^2^)	213	285	255	92.4	264	233
temp. rise upper bound (K)	99.6	126	561	91.8	473	463
experimental refs	ref 25 (275 μJ/cm^2^)	ref 63 (5.1 mJ/cm^2^, 480 K)	ref 64 (5 mJ/cm^2^, 650 K)			

Several available experimental techniques can be used for studying our proposal here: structural transitions can be probed by X-ray^65^ or electron diffraction.^64^ Ferroelectric switching can be detected via second-harmonic generation (SHG) or kelvin probe force microscopy (KPFM).^66^ Magnetic switching can be monitored using reflectance magnetic circular dichroism (RMCD)^25^ or the magneto-optical Kerr effect (MOKE).^67^

While our calculations carry some uncertainties, particularly relating to the estimation of the true band gap of semiconductors in DFT and the determination of the relaxation time τ from first-principles, we argue that the mechanism remains robust, as it is supported by band-geometric properties independent of the precise value of τ, and we expect the numbers to be representative of what is observable in a realistic sample (see Supporting Information for details). The universal properties encoded in quantum metric were recently exploited to explain optical properties of solids via the nature of their chemical bonding.^68^ As a result, we anticipate that bond engineering^69^ or weak symmetry breaking^70^ in multilayered vdW structures will significantly enhance our proposed mechanism.

## Conclusions

In this work, we demonstrated how nonlinear light–matter interactions with above-band gap light lead to structural and electronic transitions. By invoking perturbation theory in a many-body formalism, we derived the NELIC theory and revealed it as the leading-order ionic response with above-band gap light. By employing first-principles calculations, we mapped out the possible stacking configurations of bilayer CrI_3_ and MoTe_2_ and trilayer 3R–MoSe_2_, showing a rich and tunable landscape of polarization and magnetization. We then computed our reported effect, the NELIC, which causes layer sliding and ferroelectricity in CrI_3_, MoTe_2_, and 3R–MoSe_2_; in the latter, a layer-dependent substrate may be used to further tune sliding as we showed. We also showed that the FM order in CrI_3_ can be separately flipped through an NLEE. The switching is intrinsically ultrafast due to its nonthermal nature, allowing the transition to occur on the order of a picosecond, as detailed in the SI. This establishes the NELIC as a versatile, adjustable, and readily generalizable tool for the in situ manipulation of order in quantum materials.

Overall, our study lays the groundwork for nonlinear optical control of electronic excited states to achieve fast and efficient, and reversible structural and multiferroic order switching, including via subgap, or low-energy excitations,^71–75^ with broad implications for future optoelectronic and nanophotonic technologies, including high-speed optical memory and computing applications.

## Methods

All terms in eq 7 are evaluated using ab initio methods in the three 2D materials. We utilized the Vienna ab initio simulation package (VASP) to perform first-principles calculations based on density functional theory (DFT). To handle exchange–correlation interactions, we applied the generalized gradient approximation (GGA) in the form of Perdew–Burke–Ernzerhof (PBE). The core and valence electrons were, respectively, treated with the projector augmented wave (PAW) method and plane-wave basis functions, with an energy cutoff of 400 eV for CrI_3_ and 600 eV for MoTe_2_ and 3R–MoSe_2_. For DFT calculations, a Γ-centered *k* mesh was used to sample the first Brillouin zone: 7 × 7 × 1 for CrI_3_, and 11 × 11 × 1 for MoTe_2_ and 3R–MoSe_2_. To treat the d orbitals of spin-polarized Cr atoms in CrI_3_, we employed the DFT + U method, setting *U* to 3.0 eV. Noncollinear spin–orbit coupling is included in all calculations. For all three materials, we obtained the layer structures from bulk unit cells and added a 10 Å vacuum layer to avoid periodic image interactions. For each stacking **u** = (*u*_*x*_, *u*_*y*_), the in-plane coordinates were fixed while the interlayer distance was allowed to relax before subsequent calculations are performed. Relaxation was carried out until the force on each atom |*F*_*i*_| < 10^–3^ eV/Å. The out-of-plane polarization *P*_*z*_ is calculated by integrating the DFT electron density multiplied by *z* over the unit cell. We used the Wannier90 package to generate tight-binding orbitals by projecting Bloch waves in DFT calculations onto Cr-d, I-p orbitals for CrI_3_, Mo-d, Te-p orbitals for MoTe_2_, and Mo-d, Se-p orbitals for 3R–MoSe_2_. The tight-binding Hamiltonian was then used to interpolate the band structure on a finer *k* mesh (160 × 160 × 1 for CrI_3_, 320 × 320 × 1 for MoTe_2_, and 640 × 640 × 1 for 3R–MoSe_2_) to calculate the dielectric function, band energy, and density response functions, with decay rate γ_*nl*_ = γ taken to be uniformly 30 meV/*ℏ*. The *k*-mesh convergence is well-tested.

For trilayer 3R–MoSe_2_, the substrate electric field *E*_*z*_ = 0.005 V/Å was implemented as follows. For ground state energy calculations, we used VASP’s dipole correction with IDIPOL = 3, LDIPOL = TRUE., and EFIELD = 0.005. For excited state response calculations, we first performed a DFT calculation without the applied field, then added a Wannier-center-dependent on-site potential Δ*H*_*nn*_ = – *eE*_*z*_ *z*_*n*_ to each Wannier orbital *n* with center position *z*_*n*_, effectively incorporating the static field into the tight-binding Hamiltonian before computing the NELIC response.

## Supplementary Material





## Data Availability

This manuscript was previously submitted to a preprint server: Wei, H.; Kaplan, D.; Xu, H.; Li, J. Ultrafast Switchable Polar and Magnetic Orders by Nonlinear Light–Matter Interaction. 2025, arXiv:2504.04662. arXiv. https://arxiv.org/abs/2504.04662 (accessed 2026–07–05).

## Notes

Derivation of the NELIC free energy from the electron master equation; symmetry properties and Kramers–Kronig consistency of the response tensor; fluctuation–dissipation relation to linear optical conductivity; light-polarization dependence; stacking structures and symmetry analyses of bilayer CrI_3_, bilayer MoTe_2_, and trilayer 3R–MoSe_2_; switching criteria and nonconvex switching paths; comparison with direct light-ion coupling; excited-state band structures; dependence on electronic relaxation time τ; extension to femtosecond pulses and transition time scales; lattice heating estimates; and computational details (PDF)
Relaxed crystal structures of bilayer CrI_3_, bilayer MoTe_2_, and trilayer 3R–MoSe_2_ at the reference stacking (ZIP)
