# Supplementary material for: Ultrafast Switchable Polar and Magnetic Orders by Nonlinear Light–Matter Interaction
Source: ACS Nano. 2026 Aug 13;20(33):23146–56. doi: 10.1021/acsnano.5c06178 (PMC13557441; doi:10.1021/acsnano.5c06178)
Supplement: Supplementary file 1 [file nn5c06178_si_001.pdf]

Supporting Information: Ultrafast switchable polar and magnetic orders by nonlinear light-matter interaction

Haoyu Wei,[†,‡,⊥] Daniel Kaplan,[*,¶] Haowei Xu,[§,#] and Ju Li[*,‖,#]

†*Department of Applied Physics, Yale University, New Haven, Connecticut 06520, USA*

‡*Tsien Excellence in Engineering Program, Tsinghua University, Beijing 100084, China*

¶*Center for Materials Theory, Department of Physics and Astronomy, Rutgers University, Piscataway, New Jersey 08854, USA*

§*Department of Physics, City University of Hong Kong, Kowloon, Hong Kong SAR, China*

‖*Department of Materials Science and Engineering, Massachusetts Institute of Technology, Cambridge, Massachusetts 02139, USA*

⊥*Department of Materials Science and Engineering, Massachusetts Institute of Technology, Cambridge, Massachusetts 02139, USA*

#*Department of Nuclear Science and Engineering, Massachusetts Institute of Technology, Cambridge, Massachusetts 02139, USA*

E-mail: d.kaplan1@rutgers.edu; liju@mit.edu

1 Derivation of NELIC excitation energy Eq. (7)

In this section we will derive NELIC excitation energy as a function of input electric field Eq. (7) in two steps. We will first establish the excitation energy as a function of density matrix change using a many-body time-dependent DFT (TDDFT) formalism, i.e., to arrive

at Eq. (4). We will then derive perturbative expression for that density matrix change, in order to fully express the NELIC energy in terms of input light as in Eq. (7).

1.1 NELIC energy from Time-Dependent Density Functional Theory

In time-dependent density functional theory (TDDFT),[1] the time-dependent Kohn-Sham (KS) equations can be formulated as,

$$i\frac{\partial \phi_j(\mathbf{r},t)}{\partial t} = \left[-\frac{1}{2}\nabla^2 + v_{\mathrm{KS}}[n;\Phi_0](\mathbf{r},t)\right]\phi_j(\mathbf{r},t) \tag{S1}$$

where ϕ_j are the time-dependent KS orbitals, and Φ_0 is the initial many-body state e.g. a Slater determinant of single-particle orbitals $\phi_j(r)$. v_{KS} is the KS single-particle effective potential composed of three terms

$$v_{\mathrm{KS}}[n;\Phi_0](\mathbf{r},t) = v_{\mathrm{ext}}[n;\Psi_0](\mathbf{r},t) + e^2\int \mathrm{d}^3\mathbf{r}'\frac{n(\mathbf{r}',t)}{|\mathbf{r}-\mathbf{r}'|} + v_{\mathrm{xc}}[n;\Psi_0,\Phi_0](\mathbf{r},t) \tag{S2}$$

where $v_{\mathrm{ext}}[n;\Psi_0](\mathbf{r},t)$ is the external time-dependent field, and the third term v_{xc} is the exchange-correlation (xc) potential, defined to be the difference between the potential that generates density $n(\mathbf{r},t)$ in an interacting system starting in initial state Ψ_0 and the single-particle potential that generates this same density in a non-interacting system starting in initial state Φ_0. In the small excitation regime, the exchange-correlation functional $E_{\mathrm{xc}}[n(\mathbf{r})]$ and thus $v_{\mathrm{xc}}[n(\mathbf{r})]$ are empirically taken to be the same form as the ground state.[1] Similar to static DFT, the energy of the excited state can be written as a function of the KS orbitals

$$\langle H_{\mathrm{ex}}\rangle = \sum_j \rho_j\mathcal{E}_j + E_{\mathrm{xc}}[n(\mathbf{r})] - \int v_{\mathrm{xc}}(\mathbf{r})n(\mathbf{r})\mathrm{d}\mathbf{r} - \frac{e^2}{2}\iint\frac{n(\mathbf{r})n(\mathbf{r}')}{|\mathbf{r}-\mathbf{r}'|}\mathrm{d}^3\mathbf{r}\mathrm{d}^3\mathbf{r}' \tag{S3}$$

where $\mathcal{E}_j$ and ρ_j are the energy and occupation number of the j-th orbital. Since the density perturbation $\Delta n(\mathbf{r})$ is a small quantity in the perturbative regime, the increase in the many-

body total energy Eq. (S3) can be expanded as

$$\langle H_{\rm ex}\rangle - \langle H_{\rm gr}\rangle = \sum_j \mathcal{E}_j \Delta\rho_j + \sum_j \Delta\mathcal{E}_j \rho_j + \int \left(\frac{\delta E_{\rm xc}[n(\mathbf{r})]}{\delta n(\mathbf{r})}\right) \Delta n(\mathbf{r}) \mathrm{d}^3\mathbf{r} \\ - \int v_{\rm xc}(\mathbf{r})\Delta n(\mathbf{r})\mathrm{d}^3\mathbf{r} - \iint \frac{n(\mathbf{r})\Delta n(\mathbf{r})}{|\mathbf{r}-\mathbf{r}'|}\mathrm{d}^3\mathbf{r}\mathrm{d}^3\mathbf{r}' \tag{S4}$$

The third and fourth term on the right hand side of Eq. (S4) will cancel. Also, in our case, the second term cancels out with the last term. To see the latter fact, we need to evaluate the shift of eigenenergies $\Delta\mathcal{E}_j$ due to light excitation. It can be obtained by applying a first-order perturbation on the TDKS eigenequation Eq. (S1). The result is

$$\Delta\mathcal{E}_j = \langle \phi_j | \Delta H_{\rm KS} | \phi_j \rangle = \int \mathrm{d}^3\mathbf{r} \phi_j^* v_{\rm ext} \phi_j + \iint \frac{n(\mathbf{r}')\Delta n_j(\mathbf{r})}{|\mathbf{r}-\mathbf{r}'|}\mathrm{d}^3\mathbf{r}\mathrm{d}^3\mathbf{r}' \tag{S5}$$

where the first term on the right hand side of Eq. (S5) is the energy induced by the external field on the j-th KS orbit. In the present scenario where $v_{\rm ext}$ is the oscillating optical electric field potential, its time average will be zero on a timescale relevant for ionic transitions. Ignoring this oscillating term, the second term in Eq. (S5) cancels the last term in Eq. (S4). Summing up the rest, the final form of Eq. (S4) becomes

$$\langle H_{\rm ex}\rangle - \langle H_{\rm gr}\rangle = \sum_j \mathcal{E}_j \Delta\rho_j \tag{S6}$$

i.e., the excitation energy of the many-body system is equal to the excitation energy of the KS system. This is Eq. (4) in Main Text.

1.2 Derivation of the excitation energy Eq. (7)

In a quantum system with known energy levels $\mathcal{E}_m$, we assume a general master-equation time-evolution according to

$$\dot{\rho}_{nm} = \frac{1}{i\hbar}[\hat{H}, \rho]_{nm} - \gamma_{nm}\left(\rho_{nm} - \rho_{nm}^{(\mathrm{eq})}\right), \tag{S7}$$

where $\hat{H} = \hat{H}_0 + \hat{V}$ is composed of a ground-state and perturbative field Hamiltonian. $\gamma_{nm}(= \gamma_{mn})$ is a phenomenological parameter to capture the decay and dephasing of levels n and m. We know from density matrix perturbation theory[2] that under a perturbative potential $\hat{V}$ of the form

$$\hat{V} = \sum_p \hat{V}(\omega_p) e^{-i\omega_p t}, \tag{S8}$$

the second-order density matrix response is

$$\begin{aligned}\rho_{nm}^{(2)} = \sum_{\nu}\sum_{pq} & e^{-i(\omega_p+\omega_q)t} \\ & \times \left\{ \frac{\rho_{mm}^{(0)} - \rho_{\nu\nu}^{(0)}}{\hbar^2} \frac{V_{n\nu}(\omega_q)V_{\nu m}(\omega_p)}{[(\omega_{nm} - \omega_p - \omega_q) - i\gamma_{nm}][(\omega_{\nu m} - \omega_p) - i\gamma_{\nu m}]} \right. \\ & \left. - \frac{\rho_{\nu\nu}^{(0)} - \rho_{nn}^{(0)}}{\hbar^2} \frac{V_{n\nu}(\omega_p)V_{\nu m}(\omega_q)}{[(\omega_{nm} - \omega_p - \omega_q) - i\gamma_{nm}][(\omega_{n\nu} - \omega_p) - i\gamma_{n\nu}]} \right\}\end{aligned} \tag{S9}$$

where $\rho_{nm}^{(2)}$ is the 2nd order perturbation in the density matrix evaluated on its n, m-th element. The ω with double indices e.g., $\omega_{mn} = \frac{\mathcal{E}_m - \mathcal{E}_n}{\hbar}$ is the frequency gap between m and n levels, same as in Main Text; while those with single indices e.g., ω_p are frequencies of the perturbative potential $\hat{V}$.

In our case, only the DC component ($\omega_p + \omega_q = 0$) of the diagonal ($n = m$) elements are relevant. Using the ground initial state condition $f_n = \rho_{nn}^{(0)}$, and the light-matter coupling Hamiltonian $V_{mn}(\omega) = -e\mathbf{v}_{mn} \cdot \mathbf{A} = \frac{ie}{\omega}\mathbf{v}_{mn} \cdot \mathbf{E}$, the diagonal elements of the DC density

matrix, to the first non-vanishing order, is

$$\rho_{nn}^{(2)} = \frac{ie^2 f_{nl}}{\hbar^2\omega^2\gamma_{nn}} \sum_{lab\omega} \left(\frac{v_{ln}^a v_{nl}^b}{\omega_{ln} - \omega - i\gamma_{ln}} + \frac{v_{nl}^a v_{ln}^b}{\omega_{nl} - \omega - i\gamma_{nl}} \right) E_a(\omega)E_b(-\omega) \tag{S10}$$

Therefore, the excitation energy

$$\begin{aligned}\langle H_{\text{ex}}\rangle - \langle H_{\text{gr}}\rangle &= \sum_n \rho_{nn}^{(2)} \hbar\omega_n \\ &= \frac{ie^2}{\hbar\omega^2} \sum_{nl\omega ab} \left(\frac{\omega_n}{\gamma_{nn}} - \frac{\omega_l}{\gamma_{ll}} \right) \frac{f_{nl} v_{nl}^a v_{ln}^b}{\omega_{nl} - \omega - i\gamma_{nl}} E^a(\omega)E^b(-\omega)\end{aligned}$$

In our case of Bloch states, the states are also labeled by their $\mathbf{k}$ vectors. The NELIC excitation energy, after ignoring the $\mathbf{E} \cdot \mathbf{p}$ term, is given by

$$G - G_0 = \frac{ie^2 V_{\text{u.c.}}}{\hbar\omega^2} \sum_{nl\omega ab} \int_{\mathbf{k}} \left(\frac{\omega_n}{\gamma_{nn}} - \frac{\omega_l}{\gamma_{ll}} \right) \frac{f_{nl} v_{nl}^a v_{ln}^b}{\omega_{nl} - \omega - i\gamma_{nl}} E_a(\omega)E_b(-\omega), \tag{S11}$$

where as usual, $\int_{\mathbf{k}}(\cdot) = \int \frac{\mathrm{d}^d\mathbf{k}}{(2\pi)^d}(\cdot)$, and all electronic variables are implicitly labeled by $\mathbf{k}$ in addition to the explicit band indices. This is Eq. (7) in Main Text.

And the corresponding response function $\tilde{\chi}_{\text{ex}}^{ab}(\omega)$, defined by $G - G_0 = \sum_{ab,\omega} \tilde{\chi}_{\text{ex}}^{ab}(\omega)E_a(\omega)E_b(-\omega)$, is given by

$$\tilde{\chi}_{\text{ex}}^{ab}(\omega) = \frac{ie^2 V_{\text{u.c.}}}{\hbar\omega^2} \sum_{nl} \int_{\mathbf{k}} \left(\frac{\omega_n}{\gamma_{nn}} - \frac{\omega_l}{\gamma_{ll}} \right) \frac{f_{nl} v_{nl}^a v_{ln}^b}{\omega_{nl} - \omega - i\gamma_{nl}} \tag{S12}$$

2 Fluctuation-dissipation relation in the uniform decay rate γ limit

In the constant decay rate limit, the steady state of the excited electron state is one where the rate of excitation equals the rate of decay, so are the rate of energy excitation and dissipation. Therefore, the steady-state excitation energy ΔG equals the energy dissipation rate times the excitation lifetime $\tau = \gamma^{-1}$ (note that we can only do so when decay rates are equal for all energy levels, i.e. uniform γ). On the other hand, the energy dissipation comes from Joule heating, and that dissipation rate would be $\mathbf{J}(\omega) \cdot \mathbf{E}^*(\omega) = \sum_{ab} \sigma^{ab}(\omega) E_a(\omega) E_b(-\omega)$ per unit volume, where $\sigma^{ab}(\omega)$ is the optical conductivity at frequency ω. Therefore we find $\Delta G = \tau \times V_{\text{u.c.}} \sum_{ab\omega} \sigma^{ab}(\omega) E_a(\omega) E_b(-\omega)$. Plugging in the Kubo formula for optical conductivity[3,4]

$$\sigma^{ab}(\omega) = -\frac{ie^2}{\hbar} \sum_{n \neq m} \int_{\mathbf{k}} \frac{f_{nm} \omega_{nm} r^a_{nm} r^b_{mn}}{\omega_{nm} + \omega + i\tau^{-1}}, \tag{S13}$$

we get an expression for ΔG that agrees with Eq. (7) in Main Text assuming uniform γ.

3 Symmetries of response function $\tilde{\chi}^{ab}_{\text{ex}}$

Recall from Main Text Eq. (7) that our response function for excitation energy $\tilde{\chi}^{ab}_{\text{ex}}$ is

$$\tilde{\chi}^{ab}_{\text{ex}}(\omega) = \frac{ie^2 V_{\text{u.c.}}}{\hbar\omega^2} \sum_{nl} \int_{\mathbf{k}} \left(\frac{\omega_n}{\gamma_{nn}} - \frac{\omega_l}{\gamma_{ll}} \right) \frac{f_{nl} v^a_{nl} v^b_{ln}}{\omega_{nl} - \omega - i\gamma_{nl}}$$

Time reversal. Under time reversal operation $\mathcal{T}$, which consists of $\mathbf{k} \to -\mathbf{k}$ and complex conjugation $\mathcal{C}$, the energy eigenvalues are symmetric as $\omega_n(\mathbf{k}) \to \omega_n(-\mathbf{k})$, the velocity matrix elements transform as $v^a_{nl}(\mathbf{k}) \to -v^a_{ln}(-\mathbf{k})$ and the response function

$$\mathcal{T}(\tilde{\chi}^{ab}_{\text{ex}}) = \tilde{\chi}^{ba}_{\text{ex}} \tag{S14}$$

This is expected because the free energy response would be invariant if we also time-reverse ($\omega \leftrightarrow -\omega$) the applied field in the macroscopic relation $\Delta G = \sum_\omega \tilde{\chi}^{ab}_{\text{ex}} E^a(\omega) E^b(-\omega)$ (or equivalently $a \leftrightarrow b$, due to reality of the time-domain $E(t)$ field). From this result, we can see a time-reversal invariant system would have a symmetric response tensor $\tilde{\chi}^{ab}_{\text{ex}} = \tilde{\chi}^{ba}_{\text{ex}}$.

Spatial inversion. $\tilde{\chi}^{ab}_{\text{ex}}$ is invariant under spatial inversion. Under spatial inversion ($\mathcal{P}$), all quantities in Eq. (S19) remains invariant except for a k-space inversion $\mathbf{k} \to -\mathbf{k}$, which still preserves the overall sum. This is clear also from the fact that the free energy is invariant when the polarizations $\mathbf{E}$ are inverted, because it is a quadratic form in $\mathbf{E}$. This fact suggests that $\mathcal{P}$ symmetry is optional for a system to have NELIC effect.

Complex conjugation. Under complex conjugation, the frequencies ω_{nl} and Fermi factors f_{ln} remain invariant, while $v^a_{nl} \to v^a_{ln}$, and this leads to

$$\tilde{\chi}^{ab}_{\text{ex}}(\omega) = \tilde{\chi}^{ab}_{\text{ex}}(-\omega)^* \tag{S15}$$

This relation guarantees that the NELIC free energy rise calculated by $\Delta G = \sum_\omega \tilde{\chi}^{ab}_{\text{ex}}(\omega)^* E^a(\omega) E^b(-\omega)$ is real. It will also be useful in the next section where we derive the response for linearly and circularly polarized light.

Kramers-Kronig relation. The Kramers-Kronig relation is ensured by the negative imaginary part "$-i\gamma_{nl}$" in the denominator of Eq. (S12), which fundamentally originates from causality of the response in the time domain.

To mathematically show this, we note that the NELIC response tensor Eq. (S12) can be seen as a complex analytical function of ω in the upper complex plane, since all its poles $\omega = \omega_{nl} - i\gamma_{nl}$ are in the lower complex plane. Therefore, similar to the argument for Kramers-Kronig relation for linear response functions, we can also write from residue

theorem that

$$\tilde{\chi}^{ab}_{\text{ex}}(\omega) = \frac{1}{i\pi}\mathcal{P}\int_{-\infty}^{+\infty}\frac{\tilde{\chi}^{ab}_{\text{ex}}(\omega')}{\omega'-\omega}\mathrm{d}\omega' \tag{S16}$$

leading to the relation between the real and imaginary parts,

$$\mathrm{Re}\,\tilde{\chi}^{ab}_{\text{ex}}(\omega) = \frac{1}{\pi}\mathcal{P}\int_{-\infty}^{+\infty}\frac{\mathrm{Im}\,\tilde{\chi}^{ab}_{\text{ex}}(\omega)}{\omega'-\omega}\mathrm{d}\omega' \tag{S17}$$

$$\mathrm{Im}\,\tilde{\chi}^{ab}_{\text{ex}}(\omega) = -\frac{1}{\pi}\mathcal{P}\int_{-\infty}^{+\infty}\frac{\mathrm{Re}\,\tilde{\chi}^{ab}_{\text{ex}}(\omega)}{\omega'-\omega}\mathrm{d}\omega' \tag{S18}$$

which constitute the Kramers-Kronig relation for our response function.

4 Response under a linearly or circularly polarized light

In Eq. (7) in Main Text, we gave an expression for a generic input light electric field $\mathbf{E}(\omega)$, while in the examples we only used special cases of either a linearly polarized (LPL) or circularly polarized (CPL) monochromatic light. Here, we explicitly list the actual formulas used.

Since the excitation free energy

$$\langle \Delta G\rangle = \sum_{\omega}\tilde{\chi}^{ab}_{\text{ex}}(\omega)E_a(\omega)E_b^*(\omega) \tag{S19}$$

Linearly Polarized Light. For a beam linearly polarized in-plane along $\mathbf{n} = (\cos\theta, \sin\theta)$, we use

$$\begin{cases} E_x(\omega) = E_x(-\omega) \ = \ \frac{E_0}{2}\cos\theta, \\ E_y(\omega) = E_y(-\omega) \ = \ \frac{E_0}{2}\sin\theta. \end{cases} \tag{S20}$$

Substituting this into Eq. (S19), using $\tilde{\chi}^{ab}_{\rm ex}(\omega)^* = \tilde{\chi}^{ab}_{\rm ex}(-\omega)$ and the expression for intensity $I = c\varepsilon_0 \frac{E_0^2}{2}$ gives

$$\langle \Delta G \rangle_{\rm LPL} = \frac{I}{c\varepsilon_0}\Big(\mathrm{Re}\,(\tilde{\chi}^{xx}_{\rm ex}(\omega))\cos^2\theta + \mathrm{Re}\,(\tilde{\chi}^{xy}_{\rm ex}(\omega) + \tilde{\chi}^{yx}_{\rm ex}(\omega))\cos\theta\sin\theta + \mathrm{Re}\,(\tilde{\chi}^{yy}_{\rm ex}(\omega))\sin^2\theta\Big) \tag{S21}$$

Circularly Polarized Light. For a circularly polarized beam propagating along z, we take

$$\begin{cases} E_x(\omega) = \frac{E_0}{2}, & E_x(-\omega) = \frac{E_0}{2}, \\ E_y(\omega) = i\sigma\,\frac{E_0}{2}, & E_y(-\omega) = -\,i\sigma\,\frac{E_0}{2}. \end{cases} \tag{S22}$$

where $\sigma = \pm 1$ is the handedness. Using the expression for intensity $I = c\varepsilon_0 E_0^2$, the excitation free energy can be written as

$$\langle \Delta G \rangle_{\rm CPL} \;=\; \frac{I}{2c\varepsilon_0}\Big(\mathrm{Re}\,(\tilde{\chi}^{xx}_{\rm ex}(\omega)) + \sigma\,\mathrm{Im}\,(\tilde{\chi}^{xy}_{\rm ex}(\omega) - \tilde{\chi}^{yx}_{\rm ex}(\omega)) + \mathrm{Re}\,(\tilde{\chi}^{yy}_{\rm ex}(\omega))\Big) \tag{S23}$$

Generally, for an arbitrary polarization state (E_x, E_y),

$$\langle \Delta G \rangle \;=\; \Big(2\mathrm{Re}\,(\tilde{\chi}^{xx}_{\rm ex}(\omega))\,|E_x|^2 + 2\mathrm{Re}\,(\tilde{\chi}^{yy}_{\rm ex}(\omega))\,|E_y|^2 + (\tilde{\chi}^{xy}_{\rm ex}(\omega) + \tilde{\chi}^{yx}_{\rm ex}(\omega))\,E_x E_y^* + (\tilde{\chi}^{yx}_{\rm ex}(\omega) + \tilde{\chi}^{xy}_{\rm ex}(\omega))\,E_x^* E_y\Big) \tag{S24}$$

From these results, we note that the LPL response tensor is purely real. The imaginary part of the antisymmetric element $\mathrm{Im}\,(\tilde{\chi}^{xy}_{\rm ex}(\omega) - \tilde{\chi}^{yx}_{\rm ex}(\omega))$ represents the circular dichroic response, i.e., the difference in response to CPL of opposite chiralities, which is nonvanishing for time-reversal broken systems only. The other independent elements, such as $\mathrm{Im}\,(\tilde{\chi}^{xx}_{\rm ex}(\omega))$, or $\mathrm{Im}\,(\tilde{\chi}^{xy}_{\rm ex}(\omega) + \tilde{\chi}^{yx}_{\rm ex}(\omega))$ do not seem to possess physical meaning in this context.

5 Structure and Stacking symmetries in CrI_3

In Main Text we used a special stacking of CrI_3 to demonstrate NELIC-induced multiferroic switching. This structure is formed by two monolayers of CrI_3, with one being the in-plane M_z mirror image of the other. The monolayer, shown in Fig. S1(a), is made up of a Cr atom in a planar hexagonal lattice, connected by I atoms distributed out of plane. As a result, this monolayer has $C_{3z}, M_{1\bar{1}0}$ symmetry. At the stacking origin we defined ($u_x = u_y = 0$), two layers are related exactly by M_z, and the bilayer belongs to Space Group 189 by possessing bilayer C_{3z} and M_z symmetries. At other generic stackings that also do not lie at high symmetry points, the bilayer does not feature any symmetry and belongs to Space Group 1. However, spatial operations can relate one such stacking to another, producing symmetry in the stacking landscape, which also affect the existence of multiferroic switching from one stacking to another. These symmetries are what we call stacking symmetries and will be discussed below.

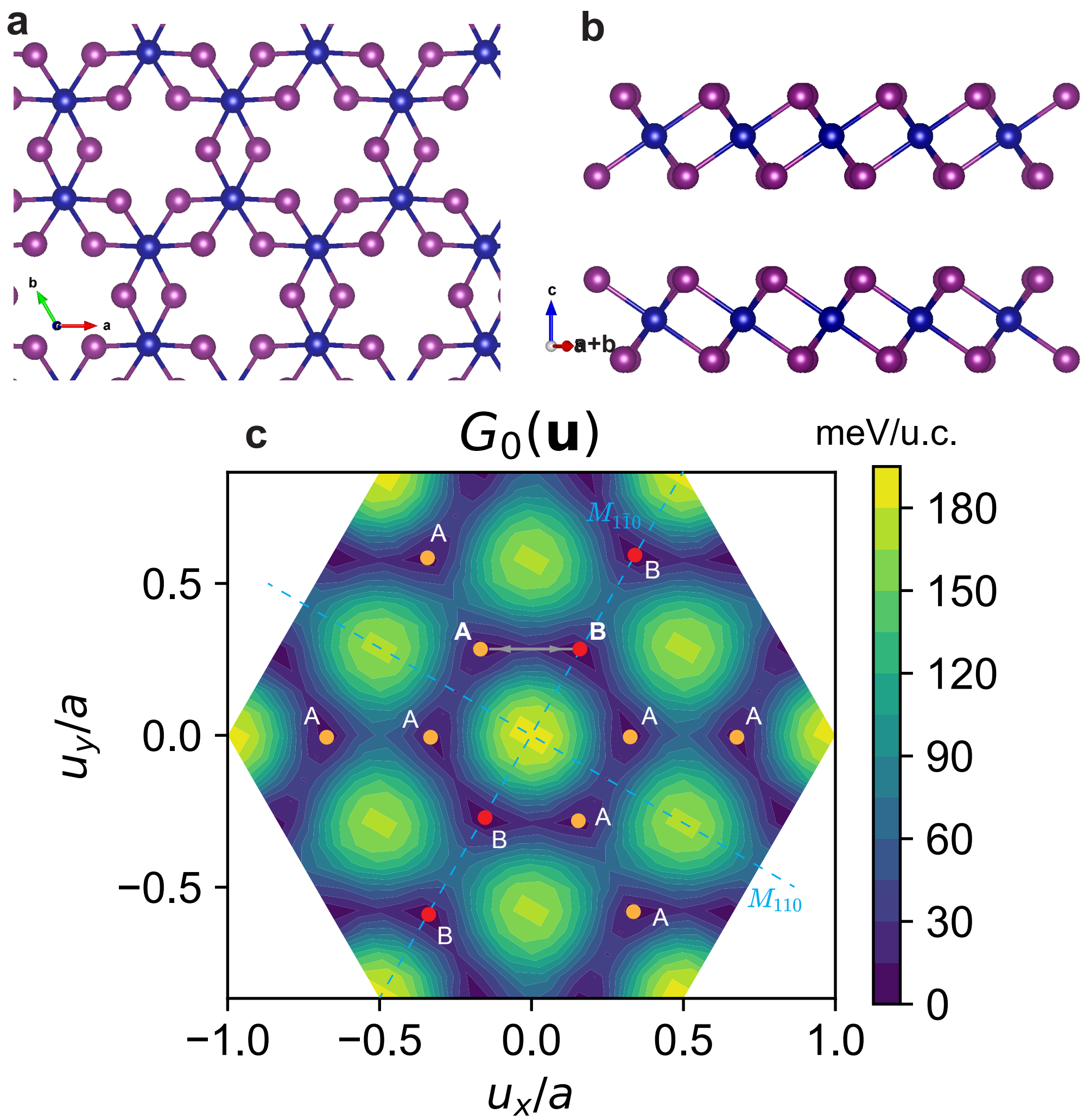

Figure S1: Structure of (a) monolayer CrI_3 and (b) bilayer M_z-CrI_3 at the stacking ($\mathbf{u} = \mathbf{0}$), showing the definition of lattice cell vectors used in our calculation on the bottom left. $\mathbf{a} + \mathbf{b}$ is the direction of the vector sum of the first two basis vectors. Cr atoms in blue; I atoms in purple. (c) Stacking energy landscape of M_z-CrI_3 showing the two symmetrically non-equivalent metastable stackings A (orange) and B (red). The grey arrow marks the unique minimum-energy path connecting A to B, which is the transition pathway used in Main Text. Mirror symmetry planes in stacking space are marked with light blue dashed lines.

The ground state energy for stackings marked by relative interlayer sliding $G_0(\mathbf{u})$, shown in Main Text Fig. 2(a), apparently has inversion symmetry C_{2z} and mirror symmetries M_{110} and $M_{1\bar{1}0}$

To understand this fact, here we introduce an analytical approach to deriving the symmetries. We denote the set of all atomic positions in a single CrI_3 layer by $L_1 = \{(x_i, y_i, z_i; n_i)\}$, the collection of atomic positions (x_i, y_i, z_i) and type (marked by n_i, which can be its atomic

number). Then, the set of atoms in a *bilayer* with sliding vector $\mathbf{u}$ would be

$$L_2(\mathbf{u}) = T(\mathbf{u}) M_z L_1 \bigcup L_1 \tag{S25}$$

where $T(\mathbf{u})$ is a translation operator that displaces the atomic set L_1 by $\mathbf{u}$, and M_z is a mirror symmetry operator with respect to a mirror plane perpendicular to $\hat{z}$) halfway between the two layers. Therefore, $T(\mathbf{u})M_z L_1$ would mean that L_1 is mirrored first (positioned at the correct z coordinate at the same time), and then translated in the $x-y$ plane by $\mathbf{u}$, to make the second layer. This second layer is then combined ("$\cup$") with the first layer L_1 to make the bilayer system, denoted as $L_2(\mathbf{u})$.

With this tool, we can analyze a few symmetries the ground state energy $G_0(\mathbf{u})$, and the excited state response function $\tilde{\chi}^{ab}_{\text{ex}}$ also has properties following from these stacking symmetries.

C_{2z} **symmetry of the bilayer.** If we apply a global $T(-\mathbf{u})M_z$ operation to $L_2(\mathbf{u})$, then the resulting structure should have the same ground state energy with the initial $L_2(\mathbf{u})$. On the other hand, the new system under operation is

$$L_2' = T(-\mathbf{u}) M_z L_2(\mathbf{u}) = L_1 \bigcup T(-\mathbf{u}) M_z L_1, \tag{S26}$$

where we used the commutator $[T(\mathbf{u}), M_z] = 0$ as $\mathbf{u}$ is in $x-y$ plane. This L_2' can be identified as $L_2(-\mathbf{u})$. Therefore, the ground state energy function has a symmetry $G_0(\mathbf{u}) = G_0(-\mathbf{u})$, i.e., an in-plane C_{2z} symmetry.

$M_{1\bar{1}0}$ **symmetry of the bilayer.** If we apply a global $M_{1\bar{1}0}$ operation to the bilayer L_2, then the resulting structure is

$$M_{1\bar{1}0} L_2(\mathbf{u}) = T(M_{1\bar{1}0}\mathbf{u}) M_z L_1 \bigcup L_1 = L_2(M_{1\bar{1}0}\mathbf{u}), \tag{S27}$$

which can be identified as $L_2(M_{1\bar{1}0}\mathbf{u})$. In the derivation of Eq. (S27), we used an internal symmetry of a single layer $M_{1\bar{1}0}L_1 = L_1$, and the commutation relation $M_{1\bar{1}0}T(\mathbf{u}) = T(M_{1\bar{1}0}\mathbf{u})M_{1\bar{1}0}$ for in-plane $\mathbf{u}$. This proves a $M_{1\bar{1}0}$ symmetry of the ground state energy function $G_0(u) = G_0(M_{1\bar{1}0}\mathbf{u})$.

M_{110} **symmetry.** M_{110} symmetry directly follows from the two symmetries (C_{2z} and $M_{1\bar{1}0}$) we proved above, due to the multiplicative relation $M_{110} = C_{2z}M_{1\bar{1}0}$.

One should note that the symmetry discussed here is distinct from the space group symmetry in a specific crystal structure, but rather the connection between different stacking structures. In M_z-CrI_3, for a generic sliding parameter $\mathbf{u}$, the structure itself can have space group symmetry as low as SG1.

Non-equivalent stackings and justification of the switching path. The stacking symmetries derived above (C_{2z}, $M_{1\bar{1}0}$, and M_{110}) partition the apparently metastable stackings of M_z-CrI_3 into symmetry-equivalent classes. As shown in Fig. S1(c), there exist two symmetrically non-equivalent configurations, between which we have studied structural switching in Main Text. It is important to note that they are not related by any of the stacking symmetries.

This non-equivalence has important consequences for NELIC-induced switching. Since NELIC energy is identical for structures related by symmetrically equivalent stackings[1], light-induced switching can only occur between non-equivalent configurations. From Fig. S1(c), it is apparent that there is essentially a unique minimum-energy path connecting A to B marked in grey, whereas paths connecting equivalent A-type stackings cannot support NELIC-driven switching. This is the path over which we consider structural switching in Main Text.

[1]The consideration of "symmetrically equivalent" should include light in the symmetry operations. For example, two structures may be related by C_{3z} but since a normally incident linearly-polarized light is nonequivalent after transformation, the NELIC response is still different.

6 Structure, stacking symmetries, and transition path in T_d-MoTe$_2$

The monolayer T_d-MoTe$_2$ crystallizes in the monoclinic space group $P2_1/\mathrm{m}$, with an in-plane mirror symmetry M_y and full inversion symmetry $\mathcal{P}$. The monolayer structure is shown in Fig. S2(a) along with the definition of axes used.

$$PL_1 = L_1 \tag{S28}$$

$$T\left(0, \frac{1}{2}\right) M_y L_1 = L_1 \tag{S29}$$

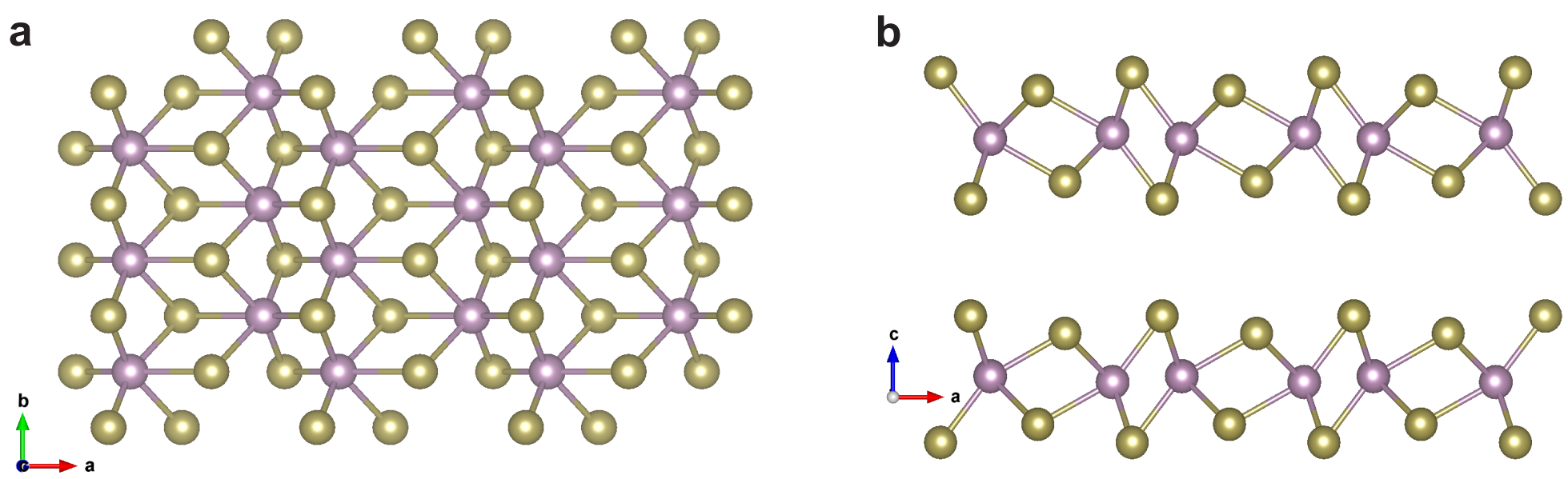

Figure S2: The crystal structure of (a) monolayer and (b) bilayer T_d-MoTe$_2$ at $\mathbf{u} = \mathbf{0}$, showing in the bottom-left corner the definition of lattice cell vectors used in our calculation. Mo atoms in pink; Te atoms in yellow.

To form a bilayer, a monolayer is stacked on top of its M_z mirror image, as shown in Fig. S2(b), and then slide the lower layer atom by $\mathbf{u} = u_x\hat{\mathbf{x}} + u_y\hat{\mathbf{y}}$, a vector we use to parametrize the stackings.

We now use the analytical tool developed in the previous section to derive stacking symmetries in M_z-T_d-MoTe$_2$. Following that notation, a bilayer system of sliding parameter $\mathbf{u}$ is written as $L_2(\mathbf{u}) = T(\mathbf{u})L_1 \bigcup M_z L_1$.

C_{2z} **symmetry.** Two structures that have sliding parameters $\mathbf{u}$ and $-\mathbf{u}(= C_{2z}\mathbf{u})$ are related by an M_z operation.

$$
\begin{aligned}
&T(-\mathbf{u})M_z L_2(\mathbf{u}) \\
=&T(-\mathbf{u})M_z M_z L_1 \bigcup T(-\mathbf{u})M_z T(\mathbf{u})L_1 \\
=&T(-\mathbf{u})L_1 \bigcup M_z L_1 \\
=&L_2(-\mathbf{u})
\end{aligned}
\tag{S30}
$$

So they have opposite out-of-plane ferroelectric polarization P_z while sharing the same energy and excitation energy response function.

M_y **mirror symmetry.** Two structures that have sliding parameters $\mathbf{u}$ and $M_y\mathbf{u}$ are related by a $T(0, \frac{1}{2})M_y$ operation.

$$
\begin{aligned}
&T(0, \frac{1}{2})M_y L_2(\mathbf{u}) \\
=&T(0, \frac{1}{2})M_y T(\mathbf{u})L_1 \bigcup T(0, \frac{1}{2})M_y M_z L_1 \\
=&T(M_y\mathbf{u})L_1 \bigcup M_z L_1
\end{aligned}
\tag{S31}
$$

M_x **symmetry.** M_x symmetry directly follows from the two symmetries (C_{2z} and M_y) proved above, due to the multiplicative relation $M_x = C_{2z}M_y$.

Choice of transition path. The stacking symmetries derived above (C_{2z}, M_y, and M_x) partition the metastable stackings of M_z-T_d-$MoTe_2$ into symmetry-equivalent classes. For NELIC-induced switching to occur, the starting and ending stackings must be symmetrically nonequivalent; otherwise, the NELIC response would be identical at both endpoints, precluding any switching.

Among all metastable stackings shown in Main Text Fig. 4(a), only two—labeled A and B—are symmetrically nonequivalent. These stackings also exhibit different ferroelectric po-

larizations ($P_z = 0$ for A vs. $P_z = -3.5$ eÅ/u.c. for B, as shown in Main Text Fig. 4(b)). We therefore select the direct sliding path A–S–B connecting these two nonequivalent stackings, which approximates the true minimal energy path for computational simplicity without affecting the qualitative behavior. Since this path transitions from zero polarization ($P_z = 0$) to a finite value, it represents a paraelectric-to-ferroelectric sliding transition.

7 Structure and Stacking Symmetry of 3R-$MoSe_2$

As a demonstration of the wide applicability of our effect, we also showed multiferroic switching induced by sliding in a trilayer 3R-$MoSe_2$ defined in Ref.[5] Here, we show the monolayer structure in Fig. S3(a) and the base trilayer 3R-$MoSe_2$ in Fig. S3(b).

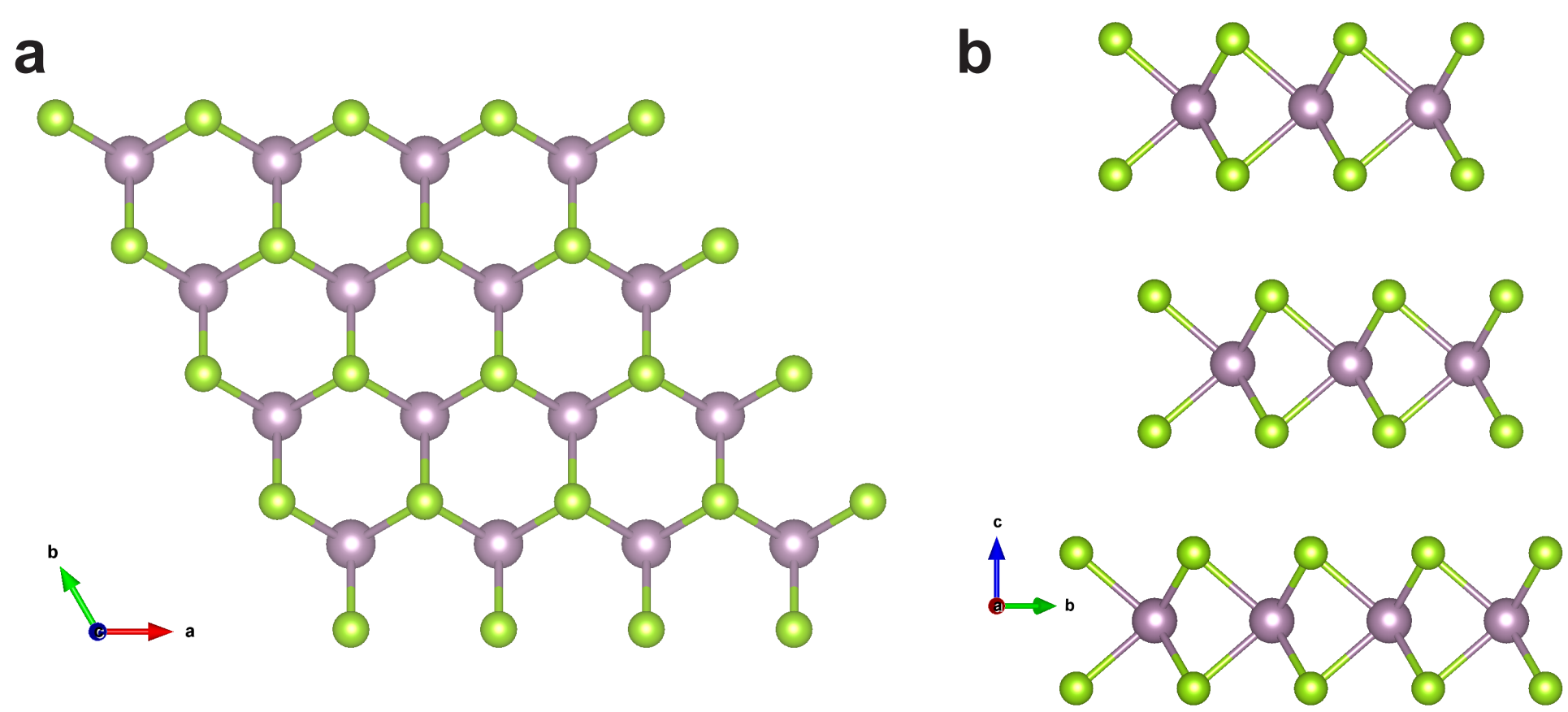

Figure S3: The crystal structure of (a) monolayer and (b) trilayer 3R-$MoSe_2$ at $\mathbf{u} = \mathbf{0}$. Mo atoms in purple; Se atoms in green.

We take this base structure as the sliding origin ($\mathbf{u} = \mathbf{0}$), and construct other stackings by sliding the top and bottom layers by equal distance but opposite directions, while keeping the middle layer fixed. In other words, we slide the lower layer by $\mathbf{u} = u_x\hat{\mathbf{x}} + u_y\hat{\mathbf{y}}$, the upper layer by $-\mathbf{u}$, and keep the middle layer fixed. Therefore we are considering a two-dimensional subspace, parametrized by $\mathbf{u}$, of all possible relative slidings of the three layers. Although a complete consideration of all stackings, which comes with a much higher computational cost, is needed to fully capture all possible paths, we believe this high-symmetry subspace is

sufficiently representative for our demonstrative purpose because it already includes both the global energy minimum (the base 3R structure) and the maximum, and preserve a $M_z M_{110}$ symmetry from both of them.

We now consider the symmetries relating different structures within this subspace. The plain monolayer has M_z, C_{3z}, M_{010} symmetries, while M_z is broken in the presence of a $+z$ substrate electric field. Following the same convention as the two sections above, 3R-$MoSe_2$ on a substrate has the following symmetries:

C_{3z} **symmetry.** Two structures that have sliding parameters $\mathbf{u}$ and $C_{3z}\mathbf{u}$ are related by an overall C_{3z} operation.

$$\begin{aligned}
&C_{3z}L_3(\mathbf{u})\\
=&C_{3z}T(-\mathbf{u})L_1 \bigcup L_1 \bigcup C_{3z}T(\mathbf{u})L_1\\
=&T(-C_{3z}\mathbf{u})L_1 \bigcup L_1 \bigcup T(C_{3z}\mathbf{u})L_1\\
=&L_3(C_{3z}\mathbf{u})
\end{aligned} \tag{S32}$$

So they have equal out-of-plane ferroelectric polarization P_z while sharing the same energy. Their NELIC response tensors $\tilde{\chi}^{ab}_{\mathrm{ex}}$ are related by a C_{3z} rotation.

M_{010} **symmetry.** Two structures that have sliding parameters $\mathbf{u}$ and $M_{010}\mathbf{u}$ are related by an overall M_{010} operation.

$$\begin{aligned}
&M_{010}L_3(\mathbf{u})\\
=&M_{010}T(-\mathbf{u})L_1 \bigcup L_1 \bigcup M_{010}T(\mathbf{u})L_1\\
=&T(-M_{010}\mathbf{u})L_1 \bigcup L_1 \bigcup T(M_{010}\mathbf{u})L_1\\
=&L_3(M_{010}\mathbf{u})
\end{aligned} \tag{S33}$$

So they have equal out-of-plane ferroelectric polarization P_z while sharing the same energy. Their NELIC response tensors $\tilde{\chi}_{\text{ex}}^{ab}$ are related by a M_{010} mirror reflection. For linearly polarized light aligned with and normal to the mirror plane, the response of the two related structures will be identical. This symmetry also maps a circularly polarized light to its opposite chirality, and since 3R-$MoSe_2$ is also nonmagnetic (time-reversal symmetric), CPL will produce identical responses, prohibiting a possible switching between related stackings.

8 Existence of switching behavior from a symmetry perspective

For switching behavior to be present in a 2D material under this mechanism, the material must have two stable states that have nonequivalent free-energy response under light. Crucially, we want to rule out identical response in these stable stackings constrained by intrinsic crystal symmetries.

For example, if a bilayer is composed of identical single-layers with in-plane mirror symmetry M_z, its two opposite stackings $\mathbf{u}$ and $-\mathbf{u}$ behave identically to NELIC. Because in this case the stackings $\pm\mathbf{u}$ are related by a global M_z, both ground state energy and excited state energy Eq. (7) are invariant under M_z, making them respond degenerately under NELIC.

Common choices such as 2H phases of TMD, and planar layers such as graphene and BN fail to satisfy the criterion of an optically controlled sliding switch because of this symmetry rule. Here, we showcase two such examples.

First, we use the example of 3R-$MoSe_2$ from Main Text, but now without the substrate electric field. The 3R-$MoSe_2$ structure, as shown in Fig. S3, has M_z symmetry in its monolayer, and therefore an overall M_z relates the trilayer at $\pm\mathbf{u}$, i.e. $M_z L_3(\mathbf{u}) = L_3(-\mathbf{u})$. With any normally incident light that does not break the M_z symmetry, the structure at two stackings respond identically to light, as shown in Fig. S4. For this reason, we assumed a substrate static electric field $E_z = 0.005 V/\text{Å}$ in Main Text to break the M_z and demonstrate

switching.

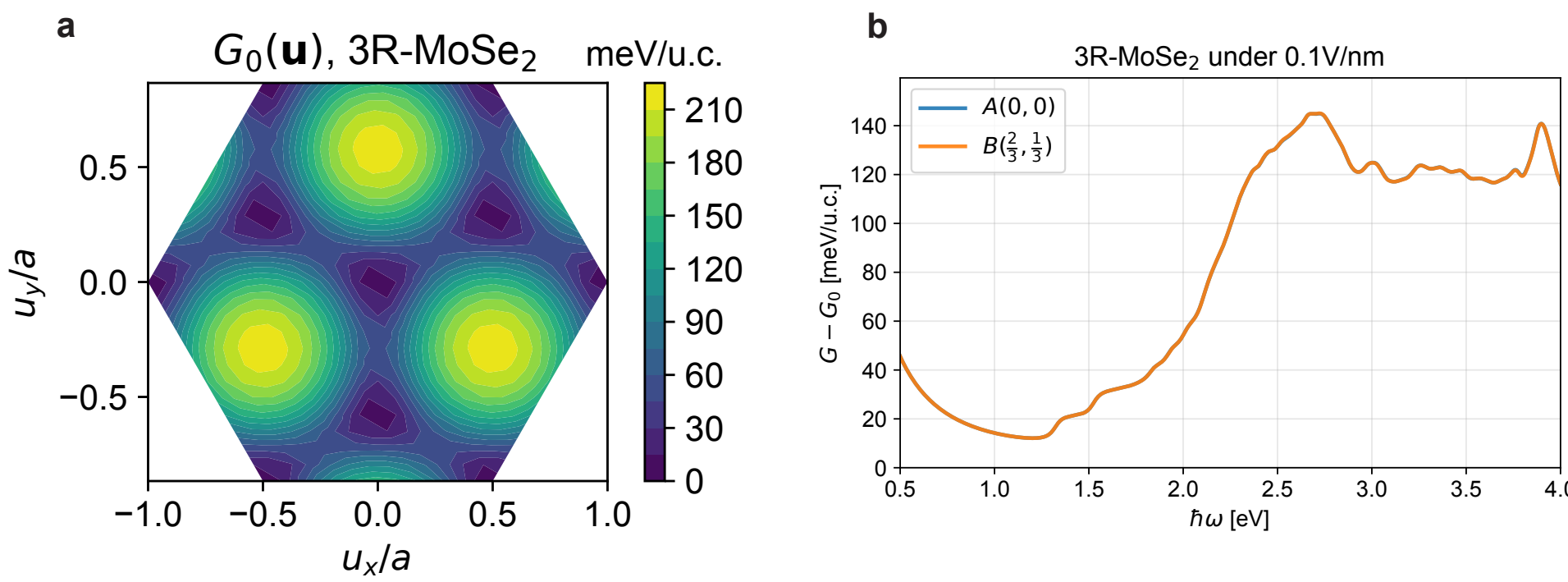

Figure S4: Absence of switching in free-standing 3R-$MoSe_2$. (a) Ground state energy landscape of 3R-$MoSe_2$ at different stackings. (b) Excitation energy response to 0.1V/nm light at the two stable stackings A and B. Linearly polarized light along y direction is used for this plot, but we see similar behavior in other polarizations as well.

We show bilayer 2H-MoS_2 as another example of symmetry-prohibited switching in Fig. S5. The two translationally nonequivalent stackings $A(\frac{2}{3},\frac{1}{3})$ and $B(\frac{1}{3},\frac{2}{3})$ are related by a global M_z symmetry, therefore the response are identical and unsuitable for light-induced switching.

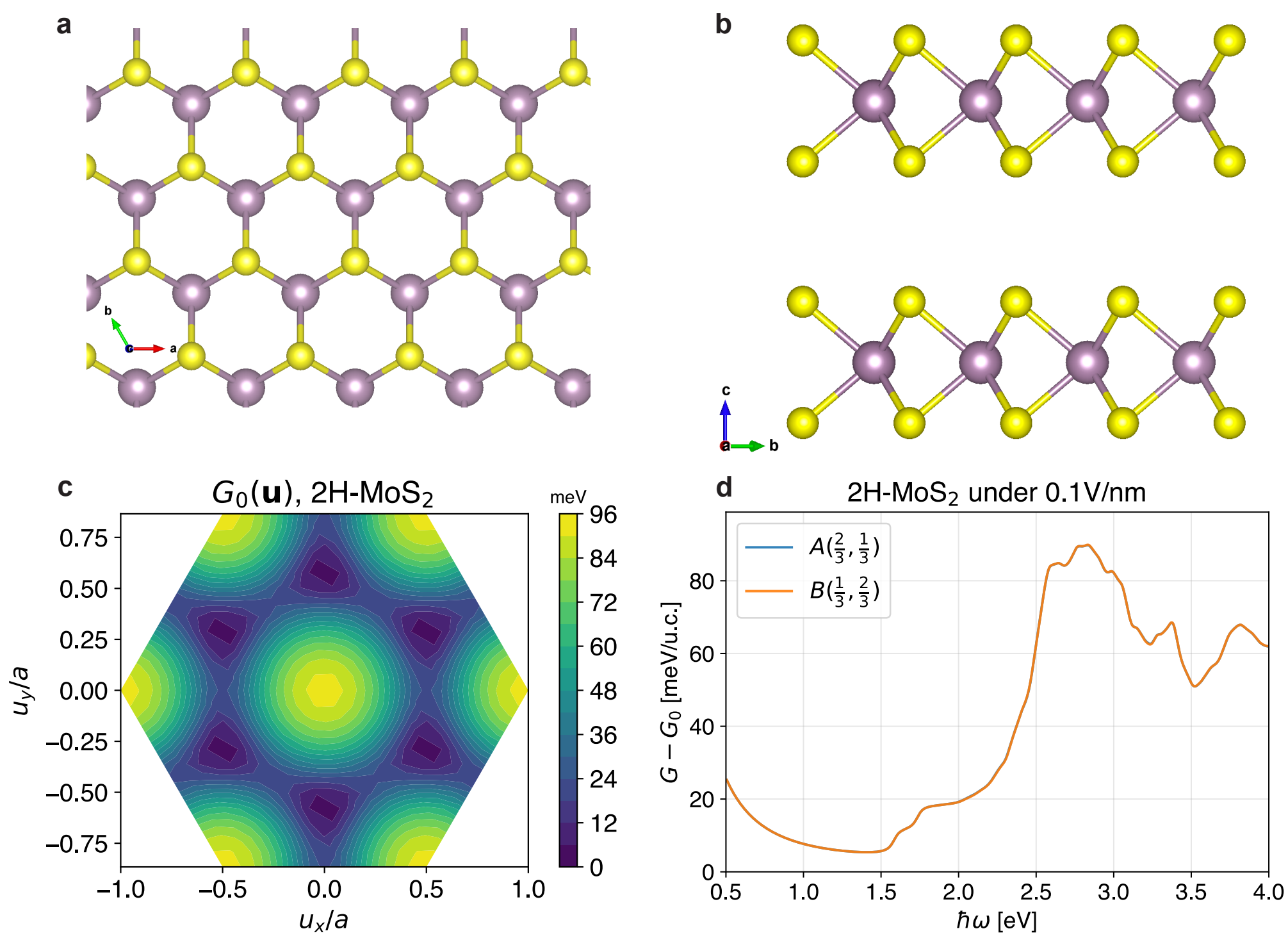

Figure S5: Absence of switching in 2H-MoS$_2$. The structure of (a) monolayer and (b) bilyer 2H-MoS$_2$. (c) Ground state energy landscape of 2H-MoS$_2$ at different stackings. (d) Excitation energy response to 0.1V/nm light at the two stable stackings A and B. Linearly polarized light along y direction is used for this plot, but we see similar behavior in other polarizations as well.

9 Determination of switching energy landscape

In Main Text Fig. 2(e)(f), Fig. 4(e)(f), and Fig. 5(e)(f), we plotted the conditions on the (ω, I) plane that would enable multiferroic switching via NELIC. Now we explain the algorithm in detail.

For each pair (ω, I), we calculate the excited state energy landscape, and take a finite difference derivative with respect to t to determine the local minima and maxima. A configuration is considered switchable from $A \to B$ if:

1. The energy minimum lies uniquely within the post-illumination interval $(S, B]$, and

2. No additional local minima or maxima exist in the preceding segment $[A, S]$.

Conversely, for $B \to A$ transitions, the conditions are mirrored:

1. The unique minimum lies within $[A, S)$, and

2. No additional local minima or maxima occur in $[S, B]$.

These logical conditions ensure that the energy profile is single-well and monotonic in the corresponding direction.

10 Convexity in the switching condition map

In Main Text Fig. 2(e)(f), we scanned through the frequency and intensity regimes of interest and the regimes that enable switching. This condition map consists of areas corresponding to smaller finite parameter regions. Most of these regions have convex outlines - corresponding to the fact that the available frequency band grows when intensity increases past a minimal critical intensity, showing the robustness to frequency variations. Other regions have an upper critical intensity, beyond which switching fails to occur. Further analysis show that they generally result from the appearance of additional energy extrema between the desired regions, halting the transition midway.

We demonstrate this with an example from M_z-CrI_3. A non-convex island of $B \to A$ switching conditions can be seen in Main Text Fig. 2(f), near 2.21eV. Here we look closely at the energy landscape, shown in Fig. S6. Note that because switching requires a unique energy minimum in the $[A, S)$ range, apart from the lower bound on switching intensity, there exists an upper bound, beyond which there is only a single minimum at S. The intermediate region, where the intricate balance between the shapes of the ground state and the NELIC excitation energy result in the unique energy minimum in the A–S region.

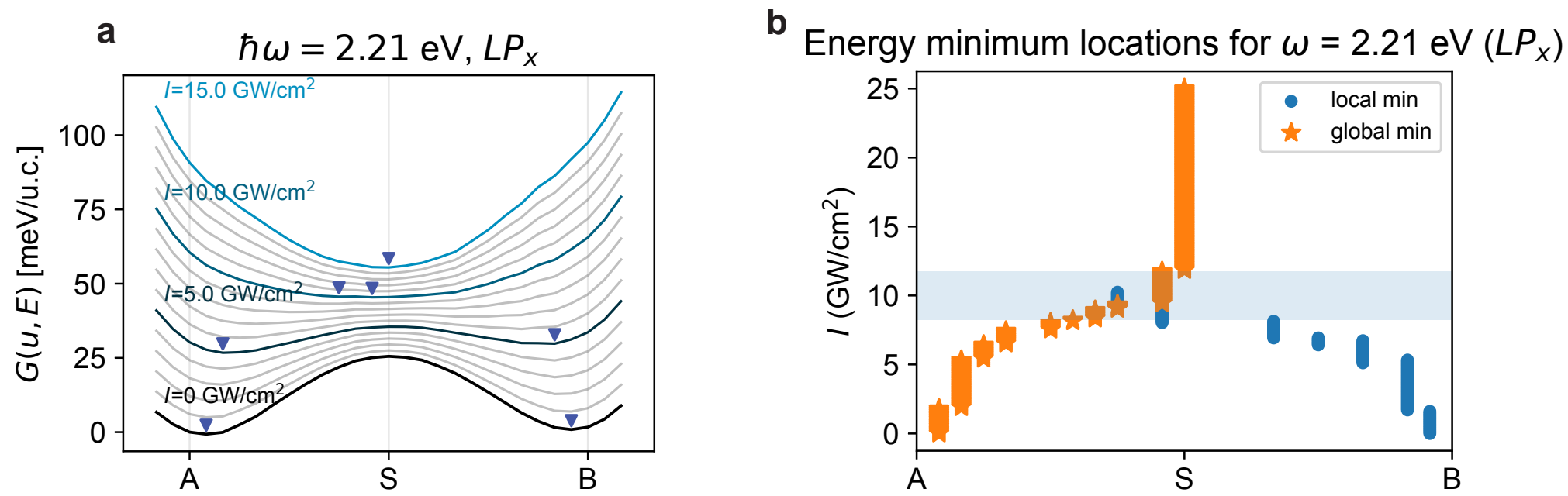

Figure S6: Switching condition in M_z-CrI_3 at $\hbar\omega$ =2.21 eV. (a) Ground and excited state energy landscape at increasing light intensity. Blue arrows show locations of energy minima. (b) The location of energy minima as a function of light intensity I. Light semitransparent blue band shows the switchable intensity range.

11 Other optical response mechanisms

The contribution introduced in Eq. (7) in the Main Text is the most dominant among other possible effects that are quadratic in $\mathbf{E}$ for inducing ionic motion and thus structural transition. We compare nonlinear excitation energy with the energy from a few other physical mechanisms.

The field interaction energy -E · p. In Main Text, we derived two terms that can contribute to the free energy change $G(\mathbf{u}, \mathbf{E}) - G_0(\mathbf{u})$: Eq. (6) and Eq. (7). Eq. (6) the energy produced by the light field interacting with the linear response (what we call the field-interaction energy). Here, we do an order of magnitude estimate for the two terms to show the relative strengths of the two terms.

For both terms, there is a common denominator can be approximated, while near resonance $|\omega - \omega_{nl}| \ll \tau^{-1}$, as

$$\frac{1}{\omega_{ln} - \omega + \frac{i}{\tau}} \approx \mathrm{P}\left(\frac{1}{\omega_{ln} - \omega}\right) - i\pi\delta(\omega_{ln} - \omega) \tag{S34}$$

Here, only the imaginary part has a non-vanishing contribution to the final free energy expression. Using also the relation $\sum_{nl} \delta(\omega_{ln} - \omega) = D(\omega)$, Eq. (6) can be estimated as

$\frac{e^2 v^2 V_{\text{u.c.}} D(\omega) E^2}{\hbar\omega^2}$, and the nonlinear excitation energy Eq. (7) is larger by a factor of $\omega\tau \gg 1$.

To validate this numerically, we compute both terms from our DFT data for M_z-CrI_3. Specifically, we calculate the optical frequency dependence of both terms in the free energy for stacking A and S, shown in Fig. S7.

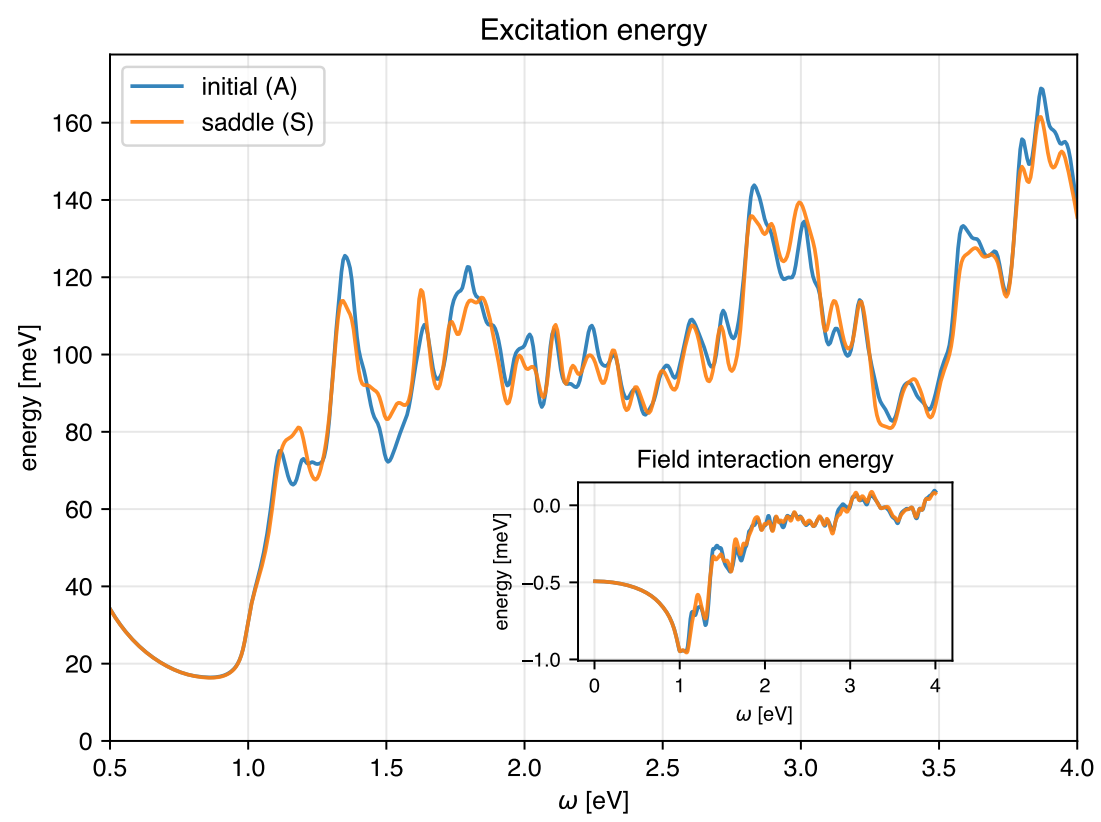

Figure S7: Comparison of nonlinear excitation Main Text Eq. (7) and the field interaction energy Eq. (6) terms at stackings A and S.

For both stackings, the nonlinear excitation energy calculated in Main Text is $\sim 10^2$ larger than the field interaction energy, as expected from the order of magnitude estimation above.

Direct light-phonon interaction. From the ground state energy curve along transition path (shown in Fig. S8), we can estimate the frequency of natural ionic oscillation and therefore the importance of direct light-phonon coupling.

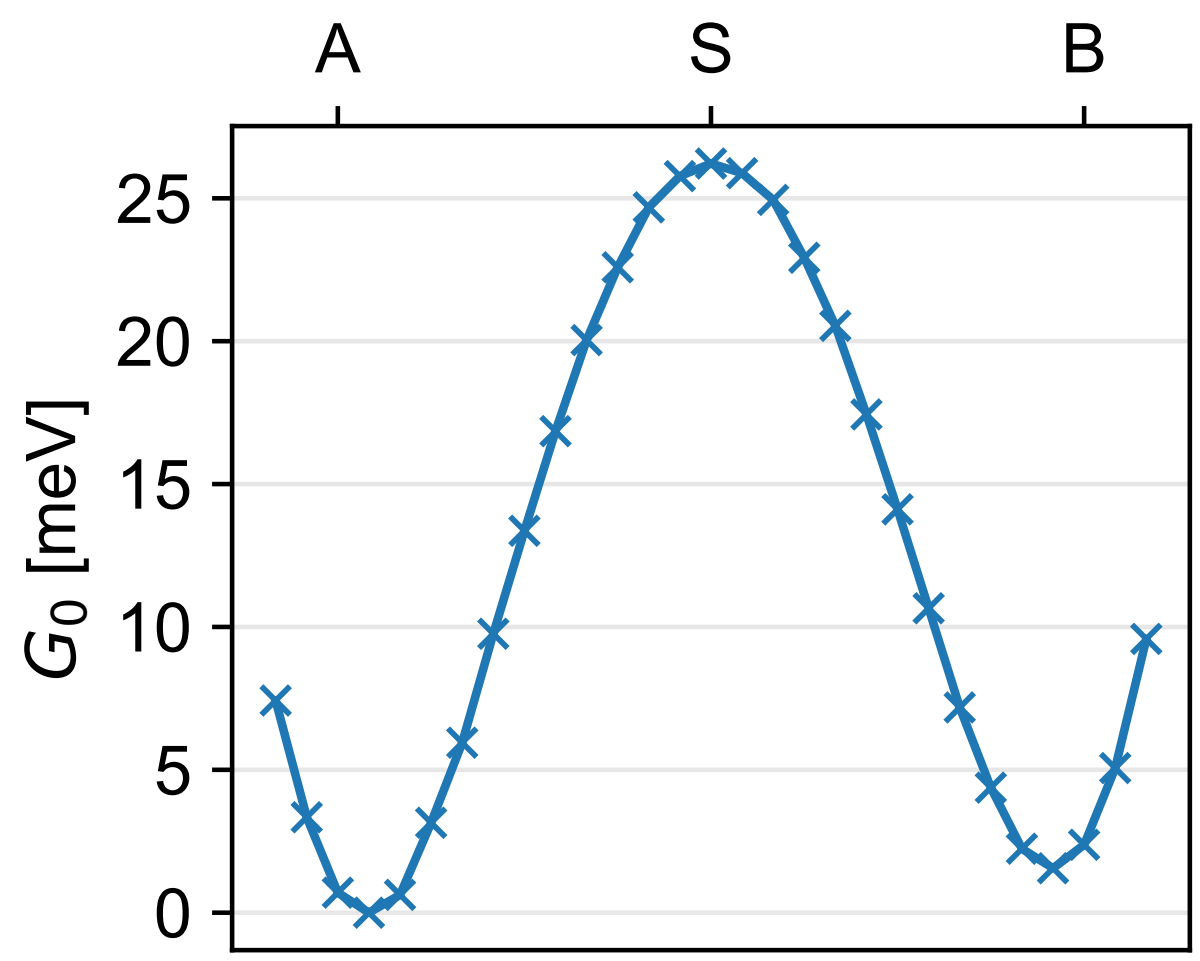

Figure S8: Ground state energy $G_0(\mathbf{u})$ of M_z-CrI_3 for stackings along A–S–B path. A, S, B are the three stackings labeled in Main Text Fig. 2.

As an example, we calculate the interlayer vibrational frequency along the transition path for stacking A. Since it is the softest degree of freedom, it is also the minimum optical phonon frequency in this system. From Fig. S8, we can quadratically fit $G_0(\mathbf{u})$ near stacking A to get $\frac{\partial^2 G_0(u)}{\partial u^2} = 204\,\mathrm{meV/Å}$. The effective mass for this vibration is half of the total mass in a unit cell, $M = 864.5$u. Hence the frequency is

$$\omega_{0,\mathrm{ion}} = \sqrt{\frac{\frac{\partial^2 G_0(u)}{\partial u^2}}{M}} = 2\pi \times 0.14\,\mathrm{THz} \tag{S35}$$

which is 3 orders of magnitude off-resonance with light used in this work $\omega \sim 2\pi \times 10^2\,\mathrm{THz}$, as expected by the relative scale of typical phonon and electronic energy levels. Due to this large off-resonance, the interlayer sliding induced is only

$$A_{0,\mathrm{ion}} = \frac{ZeE_0}{m} \frac{1}{|\omega_0^2 - \omega^2|} \sim 10^{-8}\mathrm{Å}, \tag{S36}$$

a negligible value compared to the $\sim$ 10Å required to induce a structural interlayer sliding transition. We therefore neglect this direct phonon resonance effect for the presently considered above-bandgap light.

12 Excited state band structure in M_z-CrI_3

Here we explicitly evaluate the excited state bandstructure to visualize these excitations. In Fig. S9 (b)(c), we show the excited state population for two frequencies supporting $A \to B$ and $B \to A$ transitions considered in Main Text at their respective starting stacking. The covalent nature of the relevant bands (Fig. S9 (a)) is conducive to a large quantum metric, and thus a large NELIC effect.

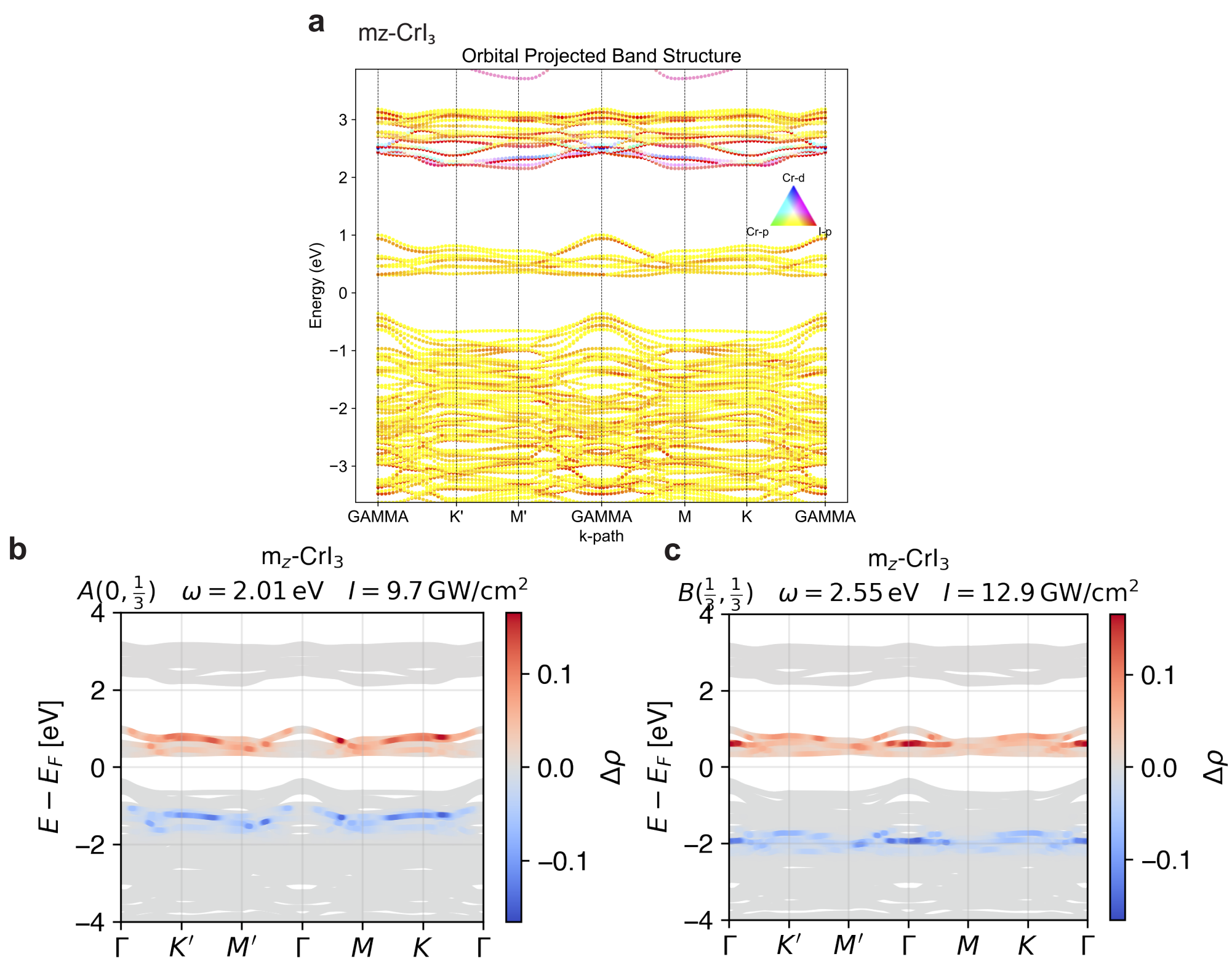

Figure S9: Orbital components and excited state in M_z-CrI_3 bandstructure. (a) A bandstructure plot of M_z-CrI_3 showing orbital constitution by color: Cr-d orbital in blue, Cr-p orbital in green, and I-p orbital in red, hybrid orbitals are mapped to color by additive color mixing. (b)(c) bandstructure plot showing electron excitation under light conditions for $A \to B$ and $B \to A$ transitions, at the respective starting stackings. The lines mark bandstructure along a high-symmetry path, with colors (in blue/red) marking the occupation number change under light. The $\mathbf{k}$-point labels Γ, M, K follow conventional definitions of high-symmetry points in a hexagonal Brillouin zone. M' and K' are time-reversed ($\mathbf{k} \to -\mathbf{k}$) counterparts of M and K.

13 Effect of the value of electronic relaxation time τ

In the calculations done in Main Text, we set the electronic relaxation time as a universal empirical constant $\tau = \frac{\hbar}{30\text{meV}}$. Here, we evaluate other possibilities of this value and its effect on the excitation energy, shown in Fig. S10.

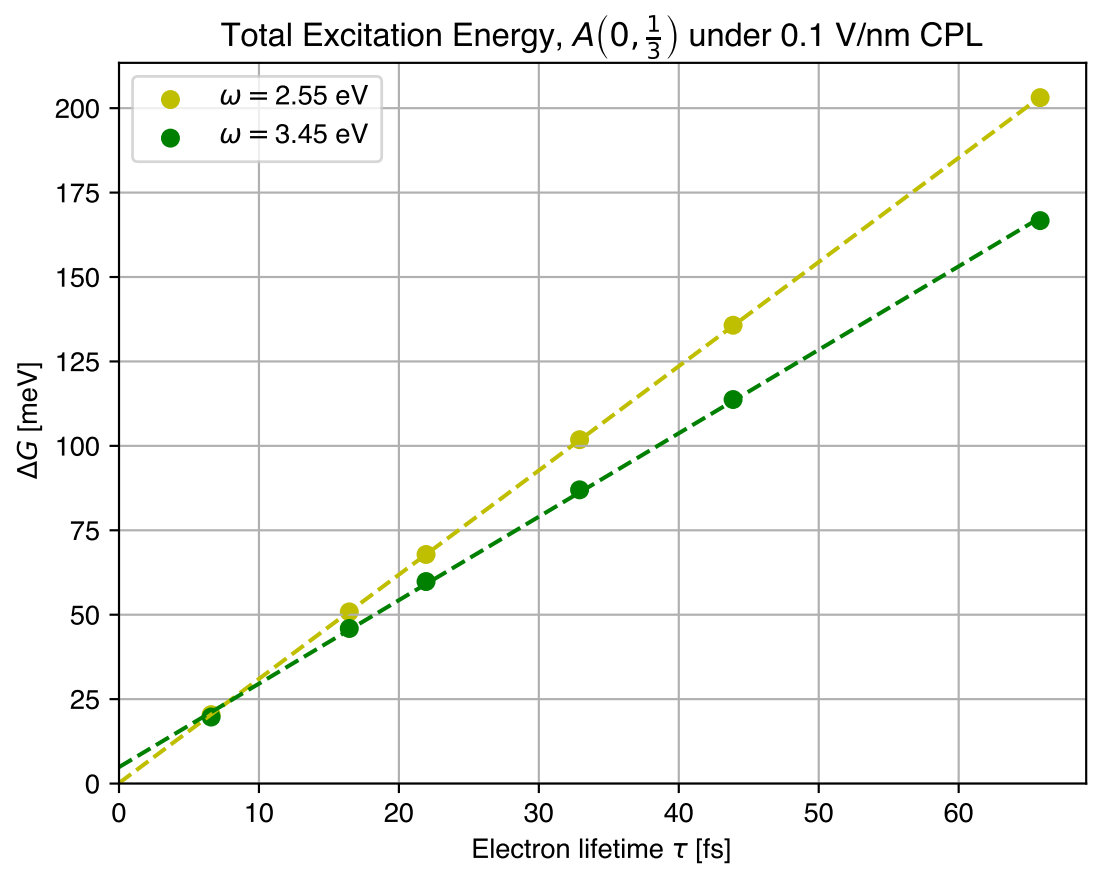

Figure S10: NELIC energy calculated for different choices of τ. Dots are data points calculated from individual DFT data. Dashed lines are linear fits.

From Fig. S10, we conclude that in the regime considered in this work, NELIC energy is to a very good approximation proportional to τ in Main Text. This proportionality is explicit in the clean-sample limit, Eq. (8), in Main Text. Practically, this implies that a change in τ value simply rescales the critical intensity for switching under continuous wave. For femtosecond pulses, as discussed in Sec. 14, NELIC response becomes essentially independent of τ for pulse durations $t_{\text{pulse}} < \tau$.

14 Effect of using short pulses, and timescales in transition

Practically in an experiment, short pulses are more often used instead of continuous waves to avoid heating-related damages. Here, we demonstrate how Eq. (7) in Main Text, originally

derived assuming continuous wave excitation, can be extended to the femtosecond pulse regime, therefore still able to achieve NELIC-induced switching.

Excitation time. When excited by a pulse, the electronic excitation number accumulated over t_{pulse} follows Fermi's golden rule

$$\rho_n = \frac{2\pi t_{\text{pulse}}}{\hbar^2} \sum_l |V_{ln}(\omega)|^2 \delta(\omega_{nl} - \omega) \tag{S37}$$

where l (n) is a valence (conduction) band index. In Sec. 13, we argued that in the regime τ can be considered large compared to the level broadening. We can then replace the delta function in (S37) as a Lorentzian with width τ to get

$$\rho_n = -\frac{it_{\text{pulse}}}{\hbar^2} \sum_l \left(\frac{f_{nl}|V_{nl}(\omega)|^2}{\omega_{ln} - \omega + i/\tau} - \frac{f_{ln}|V_{ln}(\omega)|^2}{\omega_{nl} - \omega + i/\tau} \right) \tag{S38}$$

Since $|V(\omega)|^2$ is proportional to light intensity $\sim |E|^2 \sim I$, Eq. (S38) is exactly equivalent to Eq. (8) in Main Text, replacing the factor $I \cdot \tau$ with fluence $F = I \cdot t_{\text{pulse}}$. This observation extends our NELIC scheme easily to pulses, showing that the NELIC response to pulses is insensitive to I or t_{pulse} alone, only proportional to their product F. Quantitatively, this allows us to convert our predictions of continuous waves (cw) to pulses easily: the required fluence would be the predicted cw intensities times τ, as listed in Main Text Table 1.

Temporally, after the initial electron excitation following Eq. (S38), electrons will remain in the same excited state for an extended time T_{decay} before relaxing to the ground state through radiative decay. During this time, the ions can experience the same NELIC force, so that the ferroic order switching can happen. Here, we calculate and compare the characteristic time for ions to switch ferroic state T_{switch} and the radiative decay time T_{decay}.

Furthermore, we note that even taking into account the finite spectral width (~0.1 eV) of such short pulses, the finite bandwidths in Main Text Figs. 2,4,5(e-f) suggest that NELIC-induced switching is robust to such spectral broadening (since NELIC response is additive

with respect to frequency components).

Radiative decay time. This spontaneous decay rate can be calculated with

$$\Gamma_m = \frac{4\alpha}{3c^2} \sum_{n<m} \omega_{mn} |v_{mn}|^2 \sqrt{\mathrm{Re}\, \varepsilon_r(\omega_{mn})} \tag{S39}$$

where Γ_m is the decay rate of state m, $\alpha \approx 1/137$ is the fine-structure constant, c is the speed of light in vacuum, $\varepsilon_r(\omega_{mn})$ is the relative dielectric function at frequency ω_{mn}. By an order of magnitude estimation taking $\omega_{mn} = 1\,\mathrm{eV}/\hbar$, $v_{mn} = \frac{1\mathrm{nm}}{\omega_{mn}}$, this spontaneous rate can be found to be $\Gamma \sim 1\mathrm{ns}^{-1}$. The same conclusion can be reached by evaluating (S39) for Bloch states along a high-symmetry path in Brillouin Zone of M_z-$\mathrm{CrI_3}$ at stacking A, as shown in Fig. S11.

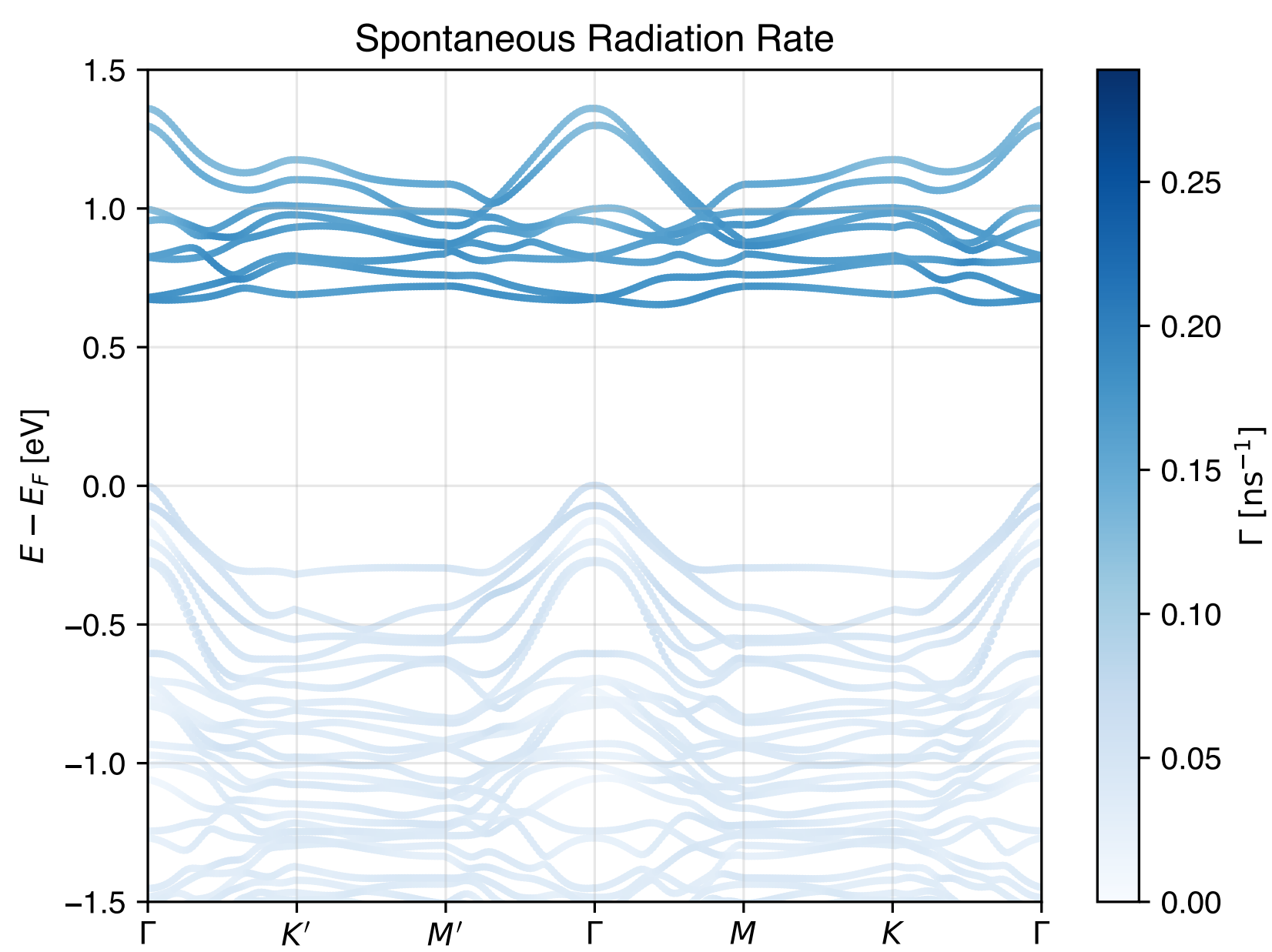

Figure S11: Spontaneous decay rate for Bloch states of M_z-$\mathrm{CrI_3}$ at stacking A, represented by colors of a bandstructure along a high-symmetry path.

Therefore, the intrinsic radiative decay time $T_{\mathrm{decay}} \sim 1\mathrm{ns}$. We note that in realistic experimental environments, exciton-phonon coupling can reduce the effective decay time to

$\gtrsim$10 ps,[6] which is still much longer than the ionic transition time $T_{\mathrm{ion}} \sim$ 1–3 ps calculated below.

Structural transition time. With the electrons being in the excited state, the system moves on a stacking-dependent potential. The time for the system to move from the initial (A) stacking to the saddle point (S) can be estimated with[7]

$$T_{\mathrm{ion}} = \int_{\mathrm{A}}^{\mathrm{S}} \frac{\mathrm{d}x}{v(x)} = \int_{\mathrm{A}}^{\mathrm{S}} \sqrt{\frac{M}{2\left(\Phi(x_{\mathrm{A}}) - \Phi(x)\right)}}\,\mathrm{d}x \tag{S40}$$

where $v(x)$ is the relative velocity between the layers, M is the effective mass per unit cell (equal to half of the total mass, 864.5 u), and $\Phi(x)$ is the ionic potential evaluated using Main Text Eq. (7). After this time T_{ion}, the structure shifts beyond the saddle point so the ionic potential does not need to remain in the excited shape: the ionic potential at electronic ground state suffices to drive the remaining ionic motion. So this is the minimum time required for electrons to stay excited.

Using M_z-CrI_3 as an example, we evaluate the structural transition time T_{ion} as a function of light intensity for both the $A \to B$ and $B \to A$ transitions, shown in Fig. S12. As the intensity increases beyond I_c, the transition time decreases monotonically due to the steeper potential gradient, but remains on the level of a few picoseconds. At $I = 1.5\,I_c$, the transition times are $T_{\mathrm{ion}} \approx 2.06$ ps for $A \to B$ and $T_{\mathrm{ion}} \approx 1.80$ ps for $B \to A$. These picosecond-scale transition times are consistent with characteristic phonon oscillation periods.

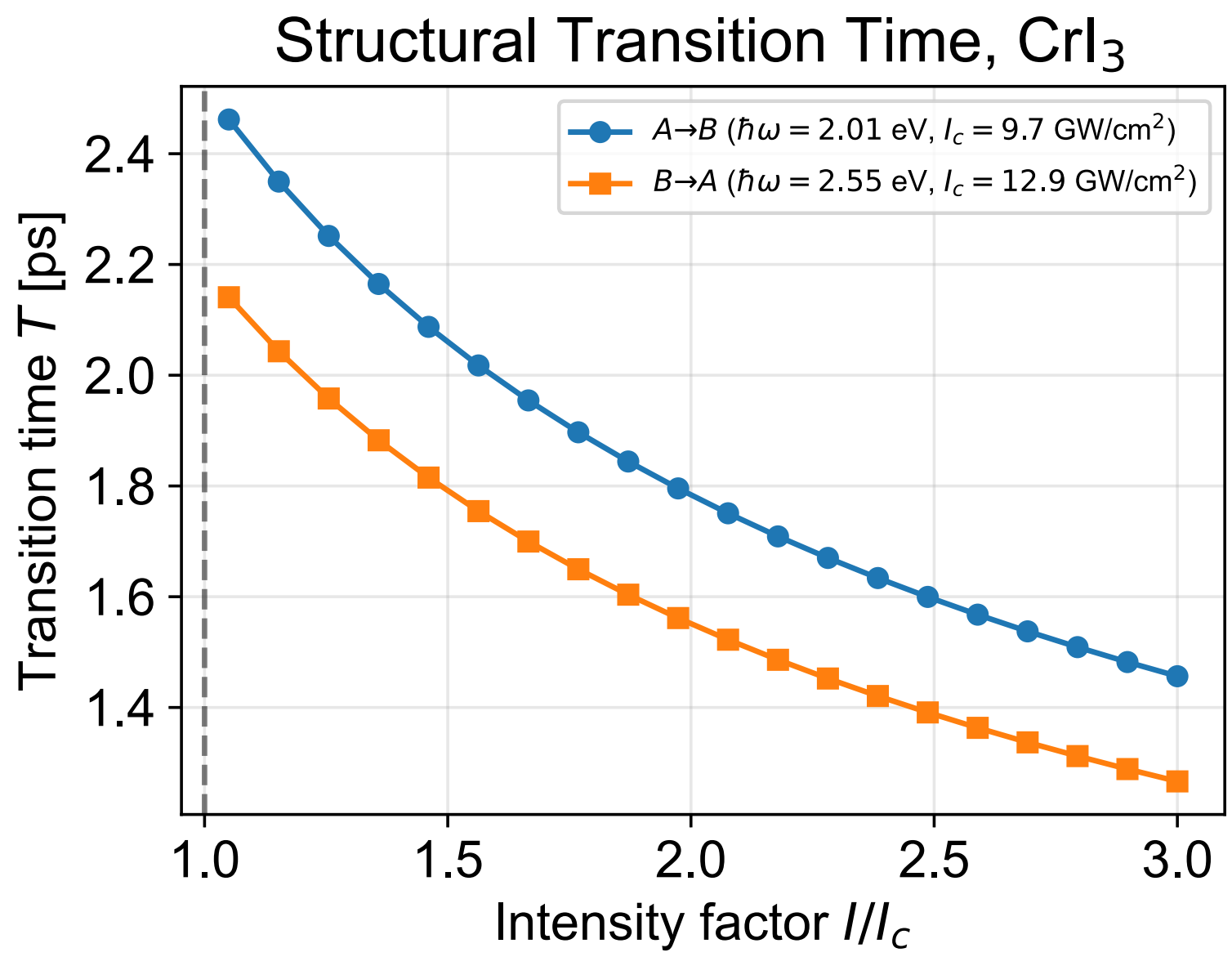

Figure S12: Structural transition time T_{ion} as a function of normalized light intensity I/I_c for M_z-CrI$_3$, for both $A \to B$ and $B \to A$ transitions. The intensity and frequency inherit values reported in Main Text.

Because the structural transition takes $\sim$ ps to happen, which is much shorter than electronic excitation decay time T_{decay}, we expect that the initial femtosecond pulse can trigger a ferroic switching despite the short pulse duration, allowing the effect to be observed under experimental achievable conditions, as we discuss in Sec. 15.

Ultrafast nature of NELIC-driven switching. The timescale analysis above establishes the ultrafast nature of the proposed switching mechanism. Three distinct timescales govern the process: (i) the optical excitation time, set by the pulse duration $t_{\text{pulse}} \sim 20$ fs, during which electrons are promoted to excited states; (ii) the ionic transition time $T_{\text{ion}} \sim 1$–3 ps (Fig. S12), during which the lattice relaxes to the new stacking configuration; and (iii) the electronic decay time $T_{\text{decay}} \sim 1$ ns, over which the excited electrons return to the ground state. The clear separation of these timescales—$t_{\text{pulse}} \ll T_{\text{ion}} \ll T_{\text{decay}}$—ensures that the structural transition completes well before the electronic excitation decays, validating our adiabatic treatment. The entire switching process from pulse arrival to structural reconfiguration thus occurs within a few picoseconds, establishing our mechanism as genuinely

ultrafast switching.

15 Thermal absorption and temperature rise

With the short pulse excitation scheme proposed in the above section, electrons are pumped to the conduction band within femtosecond time. The heating of the system only happens in the subsequent relaxation of the electrons. In this relaxation process, electrons first non-radiatively relax from their excited band to the conduction band bottom by transferring energy to phonons generating heat, and then radiatively across the bandgap without generating heat (for conductors, this step can be missing), and finally non-radiatively from the valence band top to the ground state.[8] We can derive an upper limit of the heat generated, by assuming the electrons dissipate all their excitation energies as heat except for the second step where they radiatively decay through the smallest direct bandgap. Mathematically, the released heat is

$$Q = \int_{\mathbf{k}} \left(\sum_{n} \delta\rho_{n\mathbf{k}} \hbar\omega_{n\mathbf{k}} - \sum_{n \in \text{C.B.}} \delta\rho_{n\mathbf{k}} E_g \right) = \Delta G(\mathbf{E}) - E_g \sum_{n \in \text{C.B.}} V_{\text{u.c.}} \int_{\mathbf{k}} \delta\rho_{n\mathbf{k}} \tag{S41}$$

where E_g is the global direct band gap; $n \in$ C.B. counts only electrons excited to the conduction band (excluding the negative $\delta\rho_{n\mathbf{k}}$ in the valence band), counting the number of excited electrons relaxing through the bandgap.

From Dulong-Petit law, the heat capacity of a solid is $\sim 3k_BT$ per atom.[9] The temperature rise is then

$$\Delta T = \frac{1}{3N_a k_B} \left(\Delta G(\mathbf{E}) - E_g \sum_{n \in \text{C.B.}} V_{\text{u.c.}} \int_{\mathbf{k}} \delta\rho_{n\mathbf{k}} \right) \tag{S42}$$

where N_a is the number of atoms in a unit cell. From our theory, all terms can be evaluated to derive the estimated upper bound of temperature rise due to the femtosecond excitation pulse.

Note, however, under prolonged pulses with duration exceeding the electron non-radiative

relaxation time, the temperature rise can be much higher than estimated in Eq. (S42), because electrons can relax *while* they are being pumped, creating a sustained heat source. Therefore, it is advised to use femtosecond pulses comparable to or smaller than electron relaxation time to efficiently achieve electron excitation and NELIC effect while avoiding overheating.

From Eq. (S42), we calculate the temperature rise associated with each switching and report in Table 1 in Main Text, reproduced below in Table S1. These values of $\sim 10^2$K typically stay manageable in real experiments, and will not cause permanent material damage.

Finally, we note that several factors may cause the experimentally observed temperature rise different from the values calculated to be higher or lower than estimated here.

The actual temperature rise can be lower because (a) this temperature rise value is an upper bound estimate, assuming that all radiative relaxation happens across the minimal direct bandgap; in reality, interband radiative relaxation can often happen between a wider bandgap, deeper within conduction/valence bands, so that more energy is released through the radiative channel that does not generate heat. (b) the critical intensity for sliding transition calculated in Main Text assumes a 0K condition where sliding can only happen if the BO energy surface becomes fully monotonic; while in finite temperature, sliding can happen as long as the BO energy barrier is reduced below the thermal activation energy, which can already happen at a lower light intensity. This will reduce the necessary light intensity to only a fraction, and also significantly reduce the heat.

The actual temperature rise can be higher because we simplified calculation by using solid heat capacities from Dulong-Petit law, neglecting the $\sim T^3$ region below Debye temperature; in reality the heat capacity is lower and therefore results in an order-of-unity factor correction to the temperature rise values.

Combining the two considerations, comparison with experimental fluence and temperature rise achieved suggests that the values calculated here are generally overestimations, and we therefore expect a much milder temperature rise in experiment as shown below.

In consideration of experiment, we compare these fluences or upper-bound temperature rises to previous experiments on each material. For CrI_3, Zhang *et al.* reported all-optical magnetization switching of a trilayer using circularly polarized femtosecond pulses at 2.03 eV with fluences up to $\sim$275 μJ/cm^2, a similar value to our proposed fluence, without causing structural damage.[10] For $MoTe_2$, Cheng *et al.* performed pump-probe measurements at similar wavelengths (800 nm) with fluences up to 5.1 mJ/cm^2, and estimated a maximum transient lattice temperature of $\sim$480 K.[11] For $MoSe_2$, Lin *et al.* directly tracked sub-picosecond lattice heating using MeV ultrafast electron diffraction; from the measured atomic displacement amplitudes and phonon population analysis, they reported transient lattice temperatures up to $\sim$650 K with fluences up to 5 mJ/cm^2 while pumping with 400 nm light.[12] These experimental benchmarks confirm that the relevant structural and magnetic orders remain stable and reversible under fluences and temperatures comparable to or exceeding our NELIC operating regime.

Table S1: Expected threshold intensity (under continuous wave, cw), fluence under pulses, and estimated lattice temperature rise for structural transitions considered in the Main Text, with references to representative ultrafast optical experiments on the same materials.

**Material**	M_z-CrI_3		M_z-T_d-$MoTe_2$		3R-$MoSe_2$	
**Switching**	A→B	B→A	A→B	B→A	A→B	B→A
**Photon energy** (eV)	2.01	2.55	3.48	1.94	2.47	2.80
**Wavelength** (nm)	617	486	356	639	502	443
**Intensity (cw)** (GW/cm^2)	9.7	12.95	11.6	4.2	12.0	10.6
**Fluence** (μJ/cm^2)	213	285	255	92.4	264	233
**Temperature rise upper bound** (K)	99.6	126	561	91.8	473	463
**Exp. references**	275 μJ/cm^2 (Ref.[10])		5.1 mJ/cm^2, $\sim$480 K (Ref.[11])		5 mJ/cm^2, $\sim$650 K (Ref.[12])	

16 Computational Details

All terms in Main Text Eq. (7) are evaluated using *ab initio* methods in the three 2D materials. We utilized the Vienna ab initio simulation package (VASP) to perform first-principles calculations based on density functional theory (DFT). To handle exchange-correlation in-

teractions, we applied the generalized gradient approximation (GGA) in the form of Perdew-Burke-Ernzerhof (PBE). The core and valence electrons were respectively treated with the projector augmented wave (PAW) method and plane-wave basis functions, with an energy cutoff of 400 eV for CrI_3 and 600 eV for T_d-$MoTe_2$ and 3R-$MoSe_2$. For DFT calculations, a Γ-centered k mesh was used to sample the first Brillouin zone: $7 \times 7 \times 1$ for CrI_3, and $11 \times 11 \times 1$ for T_d-$MoTe_2$ and 3R-$MoSe_2$. To treat the d orbitals of spin-polarized Cr atoms in CrI_3, we employed the DFT+U method, setting U to 3.0 eV. Non-collinear spin-orbit coupling is included in all calculations. For all three materials, we obtained the layer structures from bulk unit cells and added a 10 Å vacuum layer to avoid periodic image interactions. For each stacking $\mathbf{u} = (u_x, u_y)$, the in-plane coordinates were fixed while the interlayer distance was allowed to relax before subsequent calculations are performed. Relaxation was carried out until the force on each atom $|F_i| < 10^{-3}$ eV/Å. The out-of-plane polarization P_z is calculated by integrating the DFT electron density multiplied by z over the unit cell. We used the Wannier90 package to generate tight-binding orbitals by projecting Bloch waves in DFT calculations onto Cr-d, I-p orbitals for CrI_3, Mo-d, Te-p orbitals for T_d-$MoTe_2$, and Mo-d, Se-p orbitals for 3R-$MoSe_2$. The tight-binding Hamiltonian was then used to interpolate the band structure on a finer k mesh ($160 \times 160 \times 1$ for CrI_3, $320 \times 320 \times 1$ for T_d-$MoTe_2$, and $640 \times 640 \times 1$ for 3R-$MoSe_2$) to calculate the dielectric function, band energy, and density response functions, with decay rate $\gamma_{nl} = \gamma$ taken to be uniformly 30 meV/$\hbar$. The k-mesh convergence is well-tested.

For trilayer 3R-$MoSe_2$, the substrate electric field $E_z = 0.005$ V/Å was implemented as follows. For ground state energy calculations, we used VASP's dipole correction with `IDIPOL=3`, `LDIPOL=.TRUE.`, and `EFIELD=0.005`. For excited state response calculations, we first performed a DFT calculation without the applied field, then added a Wannier-center-dependent on-site potential $\Delta H_{nn} = -eE_z z_n$ to each Wannier orbital n with center position z_n, effectively incorporating the static field into the tight-binding Hamiltonian before computing the NELIC response.

References

1. Marques, M. A.; Maitra, N. T.; Nogueira, F. M.; Gross, E. K.; Rubio, A. *Fundamentals of Time-Dependent Density Functional Theory*; Springer, 2012; Vol. 837.

2. Boyd, R. W. *Nonlinear Optics*, 4th ed.; Academic Press is an imprint of Elsevier: San Diego, 2019.

3. Ashcroft, N. W.; Mermin, N. D. *Solid State Physics*; Holt, Rinehart and Winston: New York, 1976.

4. Komissarov, I.; Holder, T.; Queiroz, R. The Quantum Geometric Origin of Capacitance in Insulators. *Nat. Commun.* **2024**, *15*, 4621.

5. Akashi, R.; Ochi, M.; Bordács, S.; Suzuki, R.; Tokura, Y.; Iwasa, Y.; Arita, R. Two-Dimensional Valley Electrons and Excitons in Noncentrosymmetric 3R-MoS_2. *Physical Review Applied* **2015**, *4*, 014002.

6. Nie, Z.; Shi, Y.; Qin, S.; Wang, Y.; Jiang, H.; Zheng, Q.; Cui, Y.; Meng, Y.; Song, F.; Wang, X.; Turcu, I. C. E.; Wang, X.; Xu, Y.; Shi, Y.; Zhao, J.; Zhang, R.; Wang, F. Tailoring Exciton Dynamics of Monolayer Transition Metal Dichalcogenides by Interfacial Electron-Phonon Coupling. *Communications Physics* **2019**, *2*, 103.

7. Landau, L. D.; Lifshitz, E. M. *Course of Theoretical Physics*; Elsevier, 2013.

8. Chen, G. *Nanoscale Energy Transport And Conversion: A Parallel Treatment Of Electrons, Molecules, Phonons, And Photons*; Oxford University PressNew York, NY, 2005.

9. Pathria, R. K.; Beale, P. D. *Statistical Mechanics*; Academic Press, 2021; Google-Books-ID: esr2DwAAQBAJ.

10. Zhang, P.; Chung, T.-F.; Li, Q.; Wang, S.; Wang, Q.; Huey, W. L. B.; Yang, S.; Goldberger, J. E.; Yao, J.; Zhang, X. All-Optical Switching of Magnetization in Atomically Thin CrI3. *Nat. Mater.* **2022**, *21*, 1373–1378.

11. Cheng, M.; Zhong, S.; Rivas, N.; Dekker, T.; Petruk, A. A.; Gicala, P.; Pichugin, K.; Chen, F.; Luo, X.; Sun, Y.; Tsen, A. W.; Sciaini, G. Photoinduced Interlayer Dynamics in T_d-$MoTe_2$: A Broadband Pump-Probe Study. *Appl. Phys. Lett.* **2022**, *120*, 123102.

12. Lin, M.-F.; Kochat, V.; Krishnamoorthy, A.; Bassman Oftelie, L.; Weninger, C.; Zheng, Q.; Zhang, X.; Apte, A.; Tiwary, C. S.; Shen, X.; Li, R.; Kalia, R.; Ajayan, P.; Nakano, A.; Vashishta, P.; Shimojo, F.; Wang, X.; Fritz, D. M.; Bergmann, U. Ultrafast Non-Radiative Dynamics of Atomically Thin MoSe2. *Nat. Commun.* **2017**, *8*, 1745.
